# The Nephroprotective Properties of Extracellular Vesicles in Experimental Models of Chronic Kidney Disease: a Systematic Review

**DOI:** 10.1007/s12015-021-10189-9

**Published:** 2021-06-10

**Authors:** Natalia Nowak, Masayuki Yamanouchi, Eiichiro Satake

**Affiliations:** 1 Faculty of Medicine, Centre for Bioinformatics and Data Analysis, Medical University of Bialystok, Bialystok, Poland; 2 Department of Nephrology and Laboratory Medicine Faculty of Medicine Institute of Medical, Pharmaceutical and Health Sciences Graduate School of Medical Sciences, Kanazawa University, Toranomon Hospital, Nephrology Center, Tokyo, Japan; 3 Section on Genetics and Epidemiology, Research Division, Joslin Diabetes Center, Department of Medicine, Harvard Medical School, MA Boston, USA

**Keywords:** Extracellular vesicle, Exosome, Mesenchymal stem cell, MiRNA, Kidney, Chronic kidney disease, Protection, Systematic review

## Abstract

Extracellular vesicle (EV)-based therapy was hypothesized as a promising regenerative approach which has led to intensive research of EVs in various pathologies. In this study, we performed a comprehensive systematic review of the current experimental evidence regarding the protective properties of EVs in chronic kidney disease (CKD). We evaluated the EV-based experiments, EV characteristics, and effector molecules with their involvement in CKD pathways. Including all animal records with available creatinine or urea data, we performed a stratified univariable meta-analysis to assess the determinants of EV-based therapy effectiveness. We identified 35 interventional studies that assessed nephroprotective role of EVs and catalogued them according to their involvement in CKD mechanism. Systematic assessment of these studies suggested that EVs had consistently improved glomerulosclerosis, interstitial fibrosis, and cell damage, among different CKD models. Moreover, EV-based therapy reduced the progression of renal decline in CKD. The stratified analyses showed that the disease model, administered dose, and time of therapeutic intervention were potential predictors of therapeutic efficacy. Together, EV therapy is a promising approach for CKD progression in experimental studies. Further standardisation of EV-methods, continuous improvement of the study quality, and better understanding of the determinants of EV effectiveness will facilitate preclinical research, and may help development of clinical trials in people with CKD.

**Graphical Abstract:**

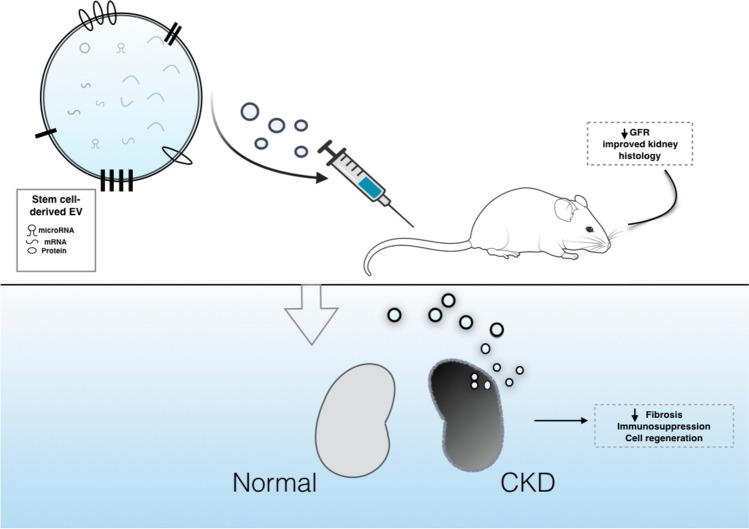

**Supplementary Information:**

The online version contains supplementary material available at 10.1007/s12015-021-10189-9.

## Introduction

Chronic kidney disease (CKD) is a serious public health concern that can lead to end-stage kidney disease (ESKD). It affects more than 0.5 billion people with a global prevalence approaching 10% [1, 2]. Despite advances in the understanding of the pathophysiology of the disease, its pharmacological treatment remains limited to supportive measures and strategies limiting CKD progression, with no therapeutic interventions specifically altering CKD cause.

CKD develops when kidney oxygen delivery is reduced. Kidney hypoxia amplifies, which is followed by an impaired regenerative capacity, induced inflammatory response, oxidative stress, cell damage, and progressive fibrosis of the kidney compartments. To counteract these processes, various pharmacological treatments have been developed. None of these, however, have been yet clinically proven to effectively alter CKD outcome [3–5]. Unfortunately, classical pharmacological approaches often ignore complexities and interconnections of overlapping disease-related mechanisms, narrowly influencing a single pathway involved in the disease pathogenesis. This limits their effectiveness and raises the risk of possible negative drug effects [2, 3]. An alternative approach, that may help overcome the bottleneck between regenerative medicine and current pharmacological treatments, relies on a targeted delivery and modulation of the disease pathways through extracellular vesicle (EV)-mediated transfer.

EVs are membranous structures released by various cell types. They encapsulate functional molecules (nucleic acids, proteins, metabolites) of the parental cells and deliver them throughout the body [6]. Because of this feature, EVs participate in intracellular communication, affecting injury and regeneration processes of the receptor cells [7, 8]. Experimental studies support the hypothesis that exogenously administered stem cell-derived EVs, are incorporated into the kidney cells and may participate in renal repair. On the other side, those EVs are free of the tumorigenic and immunogenic shortcomings of the cellular therapies; and as such, they are considered as safer and more feasible path towards future regenerative medicine [9, 10].

Recent publications have reported an evidence of significant EV therapeutic effect with regard to chronic, progressive kidney disease; and showed this effect is mediated via multiple mechanisms. However, no systematic synthesis of these data is yet available. Moreover, some of those publications pointed out discrepancies in this effect observed among study settings [10–13]. Differences in factors related to study design may have contributed to this discrepancy: (1) small study groups (2) different animal species (3) increased risk for systemic error owing to the problems in experimental design, and (4) confounding factors influencing the sensitivity of kidney regenerative processes to EV-specific stimuli. Inconsistencies in EVs in experimental CKD models may have also be caused by several factors of a biological nature, f. ex. differences in examined disease stage or by the interactions between multiple pathophysiological and protective processes, but also by different EV-protocols utilised in the studies [14] or by heterogeneous study models (i.e., different doses of EVs, xenogeneic vs allogenic EV transplant, evaluated time-points) [12, 13], and differences in the local effect of vesicular molecules [15], and thus these inconsistencies require a systematic analysis of the results.

In this report, we investigated the mechanisms by which EVs (and the molecules contained within) accelerate recovery from kidney injury in experimental CKD models. We evaluated the study design, EV-specific experiments, and analysed the active molecules contained within EVs, and their involvement in CKD pathways. Until this end, we assessed the combined protective effect in 35 studies and reported our findings following PRISMA standards [16]. Additionally, in this study we evaluated, for the first time, a combined therapeutic effect of the EV-based treatment on renal function decline estimates, and explored the potential influencing factors by stratification.

## Methods

### Search Strategy and Study Inclusion

PubMed, Cochrane, and Web of Science were systematically searched to identify all publications that have assessed EVs (small or larger EVs) in: (a) original research study in vitro and in vivo (rodent or larger animals) pre-clinical models with (b) interventional study design (c) in experimental settings of progressive kidney disease (d) where the assumed protective effects of EVs or EV-derived molecular components were the main focus of the study. The last searches were performed at the end of July, 2020. We have included only the studies which have investigated the nephroprotective properties of EVs. We have downloaded a list of studies with the following query: (((extracellular vesicle* OR EV OR exosome* OR microvesicle) AND (kidney* OR renal* OR nephro* OR CKD* OR DKD* OR UUO) AND (protection* OR repair OR prevent OR ameliorate))). The references of articles were also screened for potentially relevant studies. Additional searches were performed manually and on the Exocarta database. During screening, the articles not meeting the above criteria were excluded. Articles were selected through reading title and abstract, and if these were not informative enough, the full article was screened for eligibility. Articles were discussed between all authors before exclusion. The exclusion criteria for the studies were the following: (a) Not an interventional study (e.g. the extracellular vesicles were not directly administered as a therapy; (b) No research performed (e.g. book chapter, review article, editorial, comment, etc.); (c) Animal model of acute kidney injury (e.g. ischemia-reperfusion injury and toxic AKI models (e.g. cisplatin, glycerol, gentamycin, folic acid, lipopolysaccharide) or AKI-CKD transition), kidney stone formation; (d) No relevant data. For selected studies, the full-text articles were then extracted and further analysed. No date or location restrictions were applied. To perform the quantitative meta-analyses, we needed to exclude a) studies [40, 41, 44], which examined genetically modified EVs as miRNA delivery vectors, b) in vitro study that evaluated EV miRNA [31], c) studies that did not evaluated outcomes of GFR decline [17, 19, 35, 37], and d) a study that did not reported size of the experimental groups [44]. The remaining studies were used for extracting GFR, blood creatinine, and blood urea.

### Methods of Data Extraction

Qualitative and quantitative data were extracted for meta-analyses and/or narrative synthesis. This included information on the therapeutic intervention (including source and size of administered vesicles; route, dosing and time-point of treatment administration; vesicle content), disease model (including CKD model, animal species, strain, gender, age), and outcome results. The outcome results for meta-analyses were serum creatinine, GFR, and serum urea. All studies with outcome data and the number of experimental groups were used for extracting sample size, and mean with standard deviation (SD) of each estimate to generate standardised mean differences (SMD). For studies which reported standard error of the mean (SEM), these were converted to standard deviations (SD, where SD=√n×SEM). In a case of serial measurements, the last timepoint of the measurement for the same animals was evaluated (unless it was stated that animals were lost towards the end of the study). For studies that did not show the corresponding results in the main text, the figure calibration macro (Hessman) within ImageJ software (https://imagej.nih.gov/ij/plugins/#tools) was utilised to extract data from the graphics. The outcome results for the systematic review were recorded under the following main categories: fibrosis, inflammation, cell damage, and oxidative stress.

### Quality Assessment

To assess the methodological quality of the included experimental studies, we adapted the main criteria suggested by ISEV (MISEV, 2018). In line with these criteria, we assessed the quality of EV-included experiments by screening the concentration methods, the methodology to assess EV morphology (presence of EV enriched markers, size distribution, publication of TEM images to visualise the preparations and confirm the presence of EV population). In a formal bias analysis, we assessed study quality using the selected items from the Collaborative Approach to Meta-analysis and Review of Animal Data in Experimental Studies (CAMRADES) risk of bias checklist, with the following categories: (1) publication in peer-reviewed journal, (2) randomisation of treatment or control (3) blinded assessment of outcomes, (4) statement of compliance with regulatory requirements, and (5) statement regarding possible conflict of interest (COI).

### Methods of Analysis of EV-Based Treatment effect on Renal Function Decline

As a principle summary measure in data synthesis, we utilised the standardized mean difference (SMD) with 95% CI to combine quantitative data where the same outcome was measured using different methods/ scales. SMDs and accompanying variance were calculated for: plasma creatinine, plasma urea, and GFR. To address the issue of outcome dependance due to shared control group, we adjusted the sample sizes by dividing the reported control sample size by the number of included treatment groups (2) to equalise the weight of each group in our meta-analyses. All meta-analyses were performed for random effect models due to no a priori exclusion of studies with different experimental settings. Random-effects models were fitted using restricted maximum likelihood estimation (REML). We fitted a stratified meta-analysis model to evaluate the factors that could mediate the treatment effect. We applied the stratification criteria similar to the previous meta-analysis in CKD by Papazova et al. [16]. These included: 1) model-related factors: (a) animal species, (b) CKD model, (c) CKD etiology due to diabetes, and 2) treatment intervention-related factors: (a) treatment timing, defined as preventive if EV-based therapy was administered before clinical manifestation of CKD (for induced models, between day 0 and day 6 after induction of CKD model; for knock-out models, before clinical manifestation of disease), (b) EV dose (single vs. multiple), and (c) EV origin (allogenic vs. xenogenic). The I-squared index was primarily used to quantify the dispersion of effect size percentage of variability attributable to heterogeneity in a meta-analysis and has justified the application of the random-effects model to produce a combined therapeutic effect across heterogeneous studies. Funnel plots were used to visually assess publication study bias. In a case of visual asymmetry, the presence of small-study bias was examined using the Egger regression-based test. The trim and fill procedure was applied to adjust the results to putative publication bias. All statistical analyses were carried out with the use of Stata software version 16.1 (Stata MP, College Station, TX).

### Methods of Analysis of Vesicle-Enclosed miRNAs - Target Prediction, Data Filtering and Visualisation as Interaction Network

We have investigated the molecules encapsulated in EVs mediating their protective impact, by conducting a bioinformatic analysis. To identify targets of analysed miRNAs we have used IPA software and target scan function with the respective filters: (1) kidney tissue, and (2) an observed or predicted (with high or moderate confidence) interaction. We have build an miRNA-target network, and than subjected the targets to a network analysis. A target-target interaction network was constructed and visualised using Cytoscape software. Gene–gene interaction data were retrieved from String using StringApp package version 1.4.2 for Cytoscape. The EV microRNA database (http://bioinfo.life.hust.edu.cn/EVmiRNA/#!/) was searched for data regarding miRNA abundance in EVs from different sources.

## Results

### Search Results and Study Characteristics

**Fig. 1:**
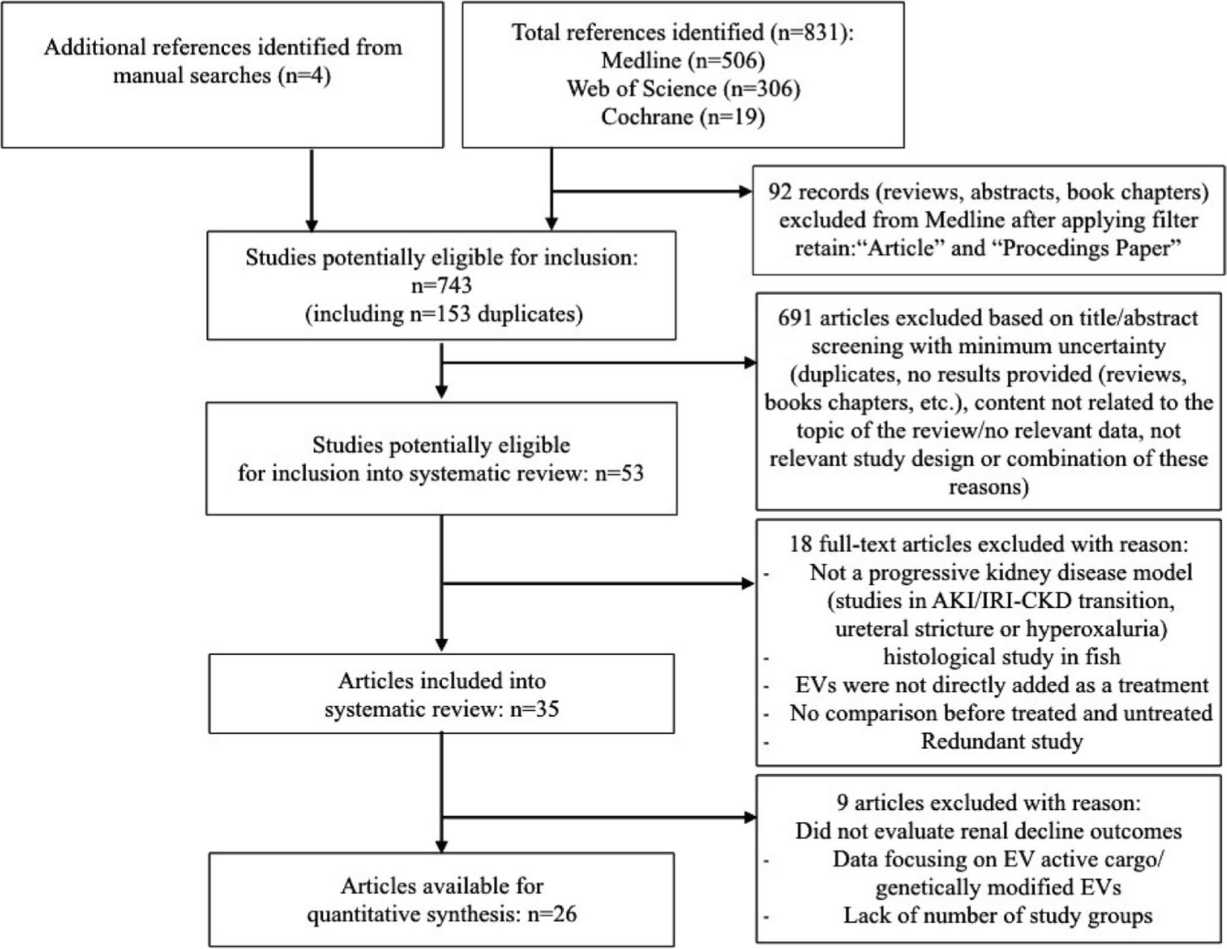
Flow chart illustrating search strategy and inclusion and exclusion of studies for systematic review and meta-analysis. Articles were selected according to criteria defined in the methods section

The flowchart of study selection for systematic review and meta-analysis is shown in Fig. 1. The initial search identified 506 articles from PubMed, 306 from Web of Science, and 19 from Cochrane. Four articles were obtained from additional searches. Among 53 full-text articles assessed for eligibility, 35 studies were included in the systematic review, including 11 studies regarding Unilateral Ureteral Obstruction (UUO), 9 studies regarding diabetic CKD settings, 7 studies regarding hypertensive (HT) CKD settings, 5 studies regarding toxic CKD, 2 studies regarding nephrectomy (Nx), and one study in Alport Syndrome. The most frequently used source of EVs were mesenchymal stem cells (MSC) derived from bone marrow, adipose tissue, or umbilical cord. EVs were also isolated from other stem cell (SC) sources, including urine, amniotic fluid (AFSC), and liver (HLSC). Other sources used to derive EVs were: cardiac progenitor cells (CPC), endothelial progenitor cells (EPC), embryonic kidney cells, and STC-like cells. In most studies, EVs were injected intravenously after the CKD model was induced.

**Fig. 2:**
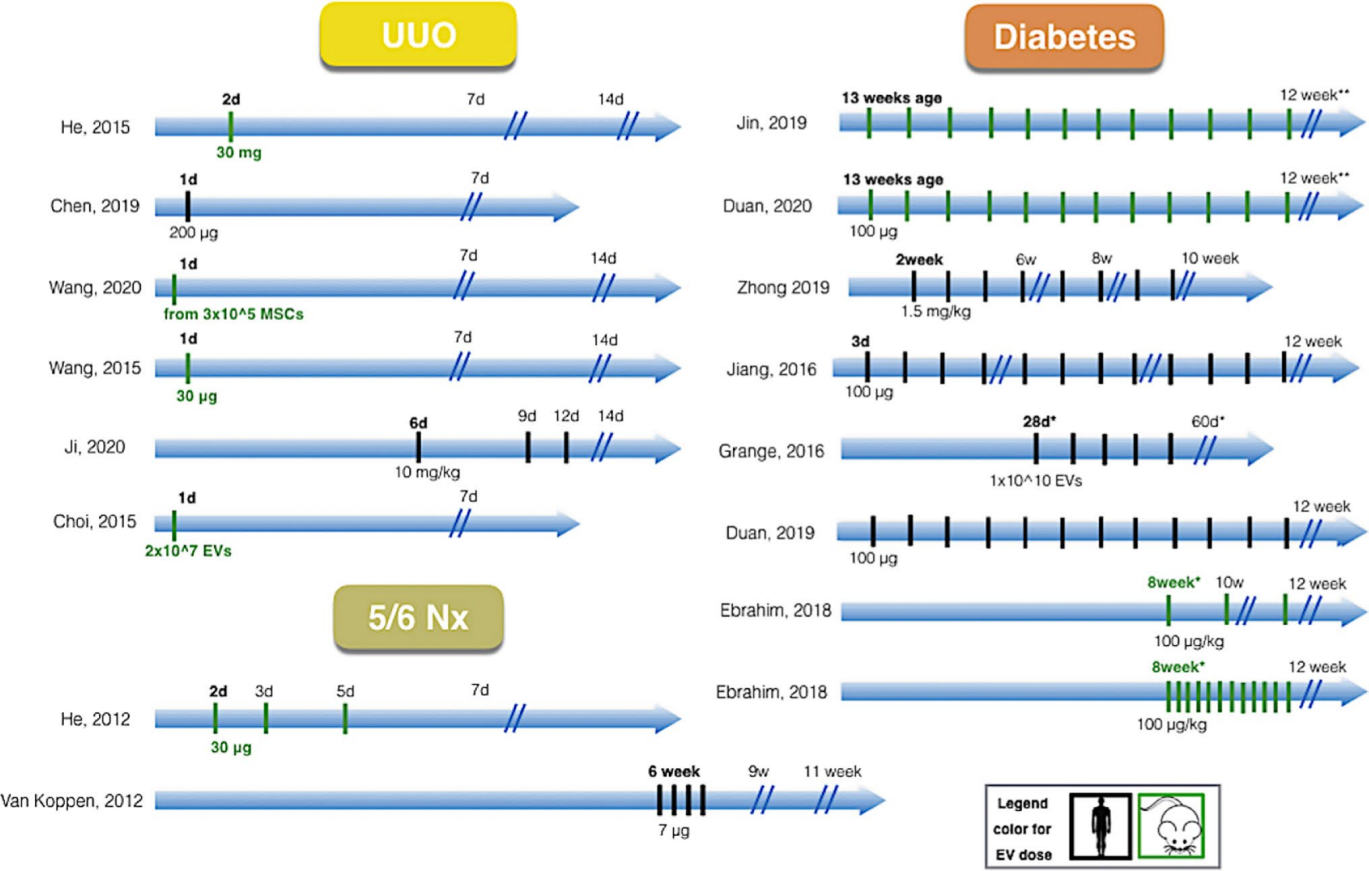
A roadmap of studies investigating extracellular vesicle (EV)-protective effect in animal CKD models. The figure shows studies in obstructive (UUO), nephrectomy (Nx) and diabetic settings of CKD, with x axis depicting time until the termination of the study. Colour of the doses represents organism of origin of EVs: black, human; green, mouse, rat. Considerable heterogeneity was visible across the included studies in terms of the experimental models used, time of EV administration, and number of EV-treatment doses Ebrahim et al. measured renal function after 2 and 4 weeks of EV administration (2 injections per day, since 8th week after diabetes onset)

**Fig. 3:**
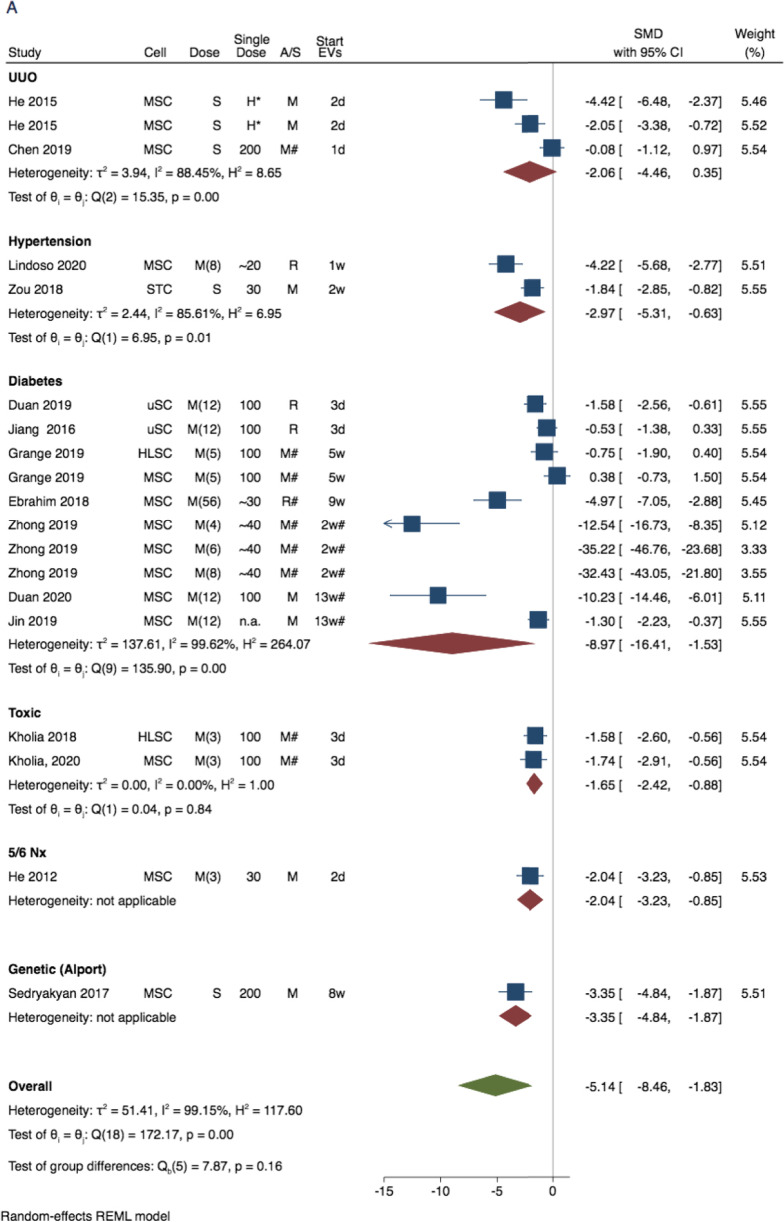

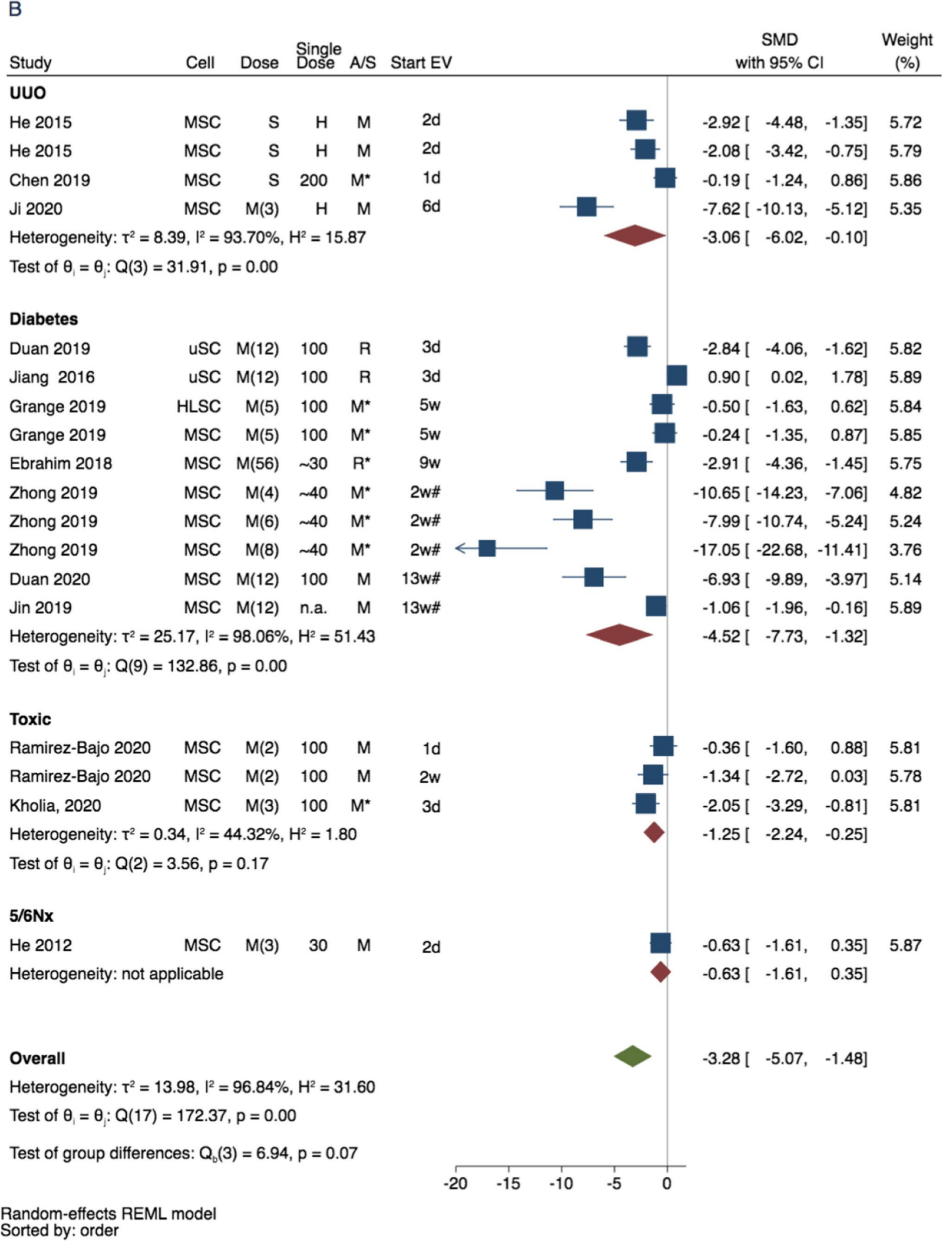
Forest plot for the therapeutic effect of extracellular vesicle (EV)-treatment on renal function decline in experimental CKD: Unilateral ureteral obstruction, hypertension, diabetes, toxic-CKD, and nephrectomy (Nx) models. Data represent SMD in (**A**) plasma creatinine and (**B**) plasma urea calculated for treaded versus non-treated comparisons of all records, excluding large animals (porcine models) and studies that did not report EV characteristics (Analyses for all study cohort is show in Supplementary Fig. 3). Abbreviations: 95% confidence interval (95% CI). RE, random effect. Cell indicates cell of EV origin, dose indicates approximate (recalculated as in Table 1) single dose in µg protein, A/S indicates animal species, start EV theraphy indicates days or weeks since model induction

**Fig. 4:**
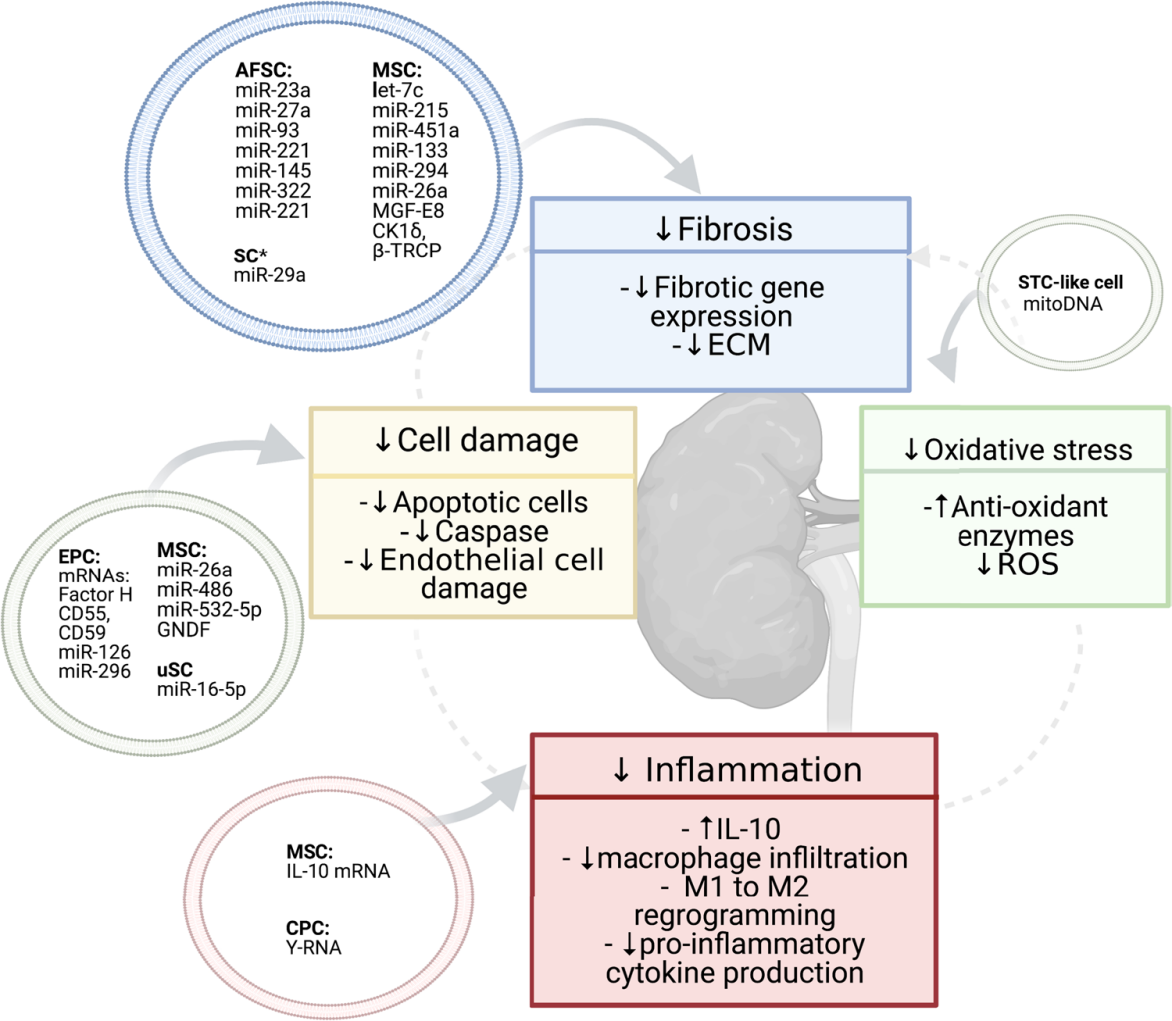
Regulatory mechanisms of extracellular vesicle-encapsulated content: a representation of the investigated molecules encapsulated in mesenchymal stem cell (MSC)-EVs, amniotic fluid stem cells (AFSC)-EVs, cardiac progenitor cell (CPC)-EVs, endothelial progenitor cell (EPC)-EVs, urine stem cell (uSC)-EVs, and kidney STL-like cell-EVs, based on involvement in fibrosis, inflammation, cell damage, and oxidative stress. Details of the study design of the EV effector molecules are presented in Table 2. The figure was created with the use of Biorender

**Fig. 5:**
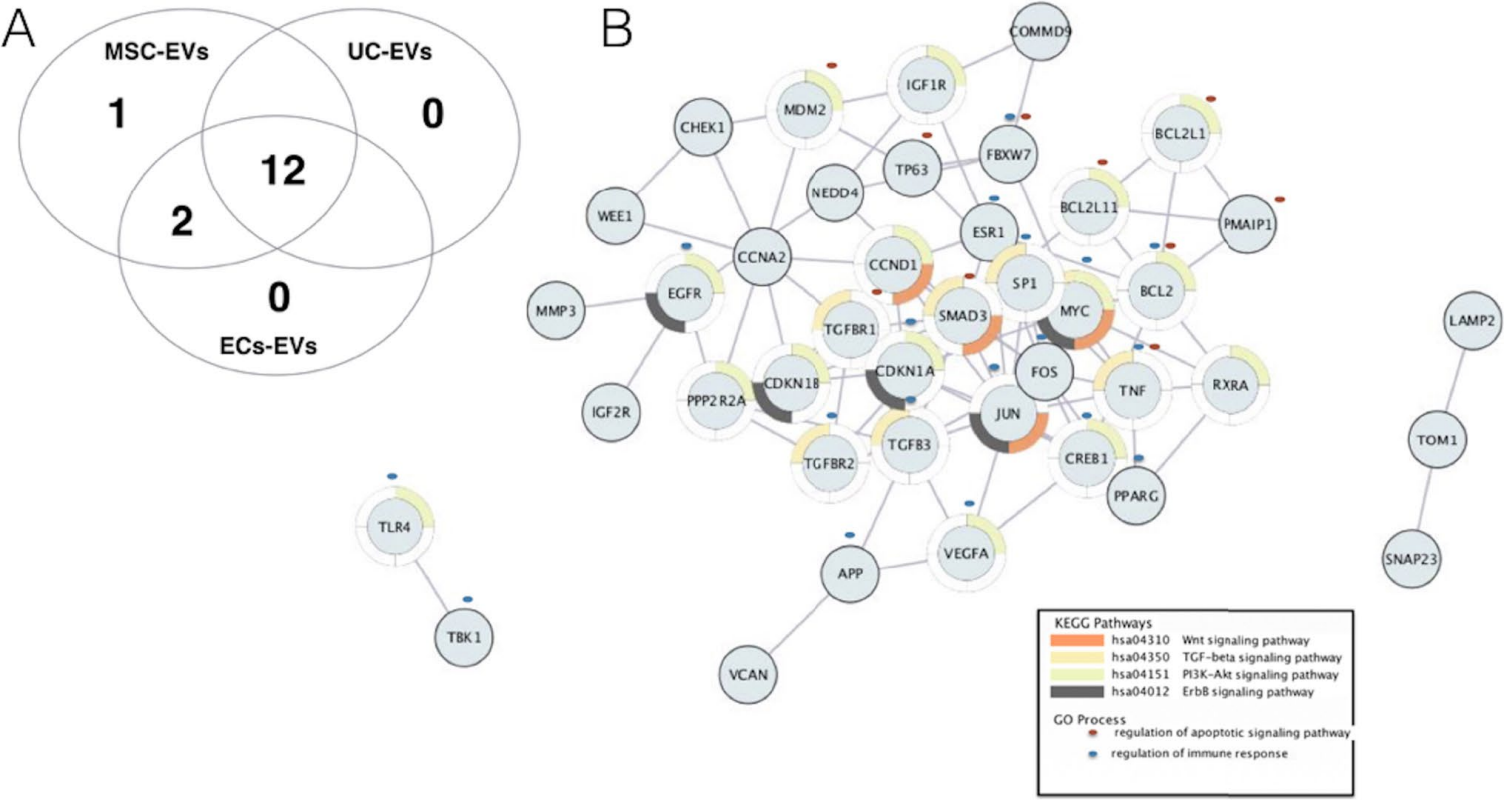
Regulatory mechanisms of extracellular vesicle-encapsulated (EV) content: bioinformatic analysis of protective miRNAs: (**A**) Venn diagram illustrating an overlap of the protective miRNAs in EVs from different cell sources. The miRNA expression data were obtained from the EV miRNA database at http://bioinfo.life.hust.edu.cn/EVmiRNA): only miRNAs with expression above 100 rpm threshold were included. (**B**) miRNA Target–target interaction network. The nodes indicate target genes, and the edges represent interactions. Only the interactions with a high evidence, ie. evidence score above 0.7 were retained. The molecules without interactions in the PPI network were removed from the figure. Colour in a circle represents different KEGG pathways implicated in kidney fibrosis. Blue, and purple marks represent specific GO process - regulation of immune response, and cell death, respectively

The details of the design of the included articles (and records within), classified according to the utilised CKD model (UUO, HT, diabetes and other) is summarised in Table 1, while the complexity of the therapeutic interventions between different CKD settings is illustrated in Fig. 2. The included studies showed considerable heterogeneity with respect to quality and experimental design, f.ex. CKD model, EV origin, time-points of EV administration and dose.

**Table 1:** Details of the design of included studies/cohorts within. Studies are classified based on CKD model as: unilateral ureteral obstruction (UUO), hypertension, diabetes and other. Characteristic of the EV intervention, disease/animal model, timepoints evaluated, and main study findings are outlined

CKD model	Study	EV source	Dosing	Adm-route	Dose	CM	EV size	Markers	Species/Model	Sex	EV Adm	End	Main findings
UUO	He [27]	MSCs (mouse) bone marrow	S	IV	30 mg	UC	~ 100 nm (TEM)	n.a.	C57BL6/J mouse (UUO), In vitro (human TECs, TGF-β1)	n.a.	2d	1w 2d	EVs alleviated EMT, and preserved renal function.
UUO	He [27]	MSCs (mouse) bone marrow	S	IV	30 mg	UC	~ 100 nm (TEM)*	n.a.	C57BL6/J mouse (UUO), In vitro (human TECs, TGF-β1)	n.a.	2d	2w 2d	EVs alleviated EMT, and preserved renal function.
UUO	Wang [39]	MSCs (mouse) bone marrow	S	IV	30 μg	UC	n.a.	n.a. (MSC-markers)	C57BL/6 mouse, (UUO), In vitro (human TECs, TGF-β1)	n.a.	1d	1w	EVs improved fibrosis, reduced tubular damage, and preserved renal function.
UUO	Wang [39]	MSCs (mouse) bone marrow	S	IV	30 μg	UC	n.a.	n.a. (MSC-markers)	C57BL/6 mouse, (UUO), In vitro (human TECs, TGF-β1)	n.a.	1d	2w	EVs improved fibrosis, reduced tubular damage, and preserved renal function.
UUO	Wang [38]	MSCs (rat) bone marrow,: younger rat	S	IV	~30 μg* (from 3x10^5 cells)	UC	n.a.	n.a (MSC-markers)	Fisher 344 rat (UUO), In vitro (h TECs, TGF-β1)	M	1d	1w	EVs improved tubulointerstitial injury. EVs failed to preserve SCr, preserved BUN.
UUO	Wang [38]	MSCs (rat) bone marrow: younger rat	S	IV	~30 μg	UC	n.a.	n.a. (MSC-markers)	Fisher 344 rat (UUO), In vitro (h TECs, TGF-β1)	M	1d	2w	EVs improved tubulointerstitial injury, failed to preserve SCr, minimally decreased BUN.
UUO	Wang [38]	MSCs (rat) bone marrow, older rat	S	IV	~30 μg	UC	n.a.	n.a. (MSC-markers)	Fisher 344 rat (UUO)	M	1d	1w	EVs failed to preserve tubulointerstitial injury, SCr and BUN.
UUO	Wang [38]	MSCs (rat) bone marrow, older rat	S	IV	~30 μg	UC	n.a.	n.a. (MSC-markers)	Fisher 344 rat (UUO)	M	1d	2w	EVs failed to preserve tubulointerstitial injury, SCr and BUN.
UUO	Chen [10]	MSCs (human) adipose tissue	S	IV	200 μg	UC	30-150 nm (DLS)	CD9, CD63, CD81	Nude mice* (UUO), In vitro (human EC)	M	1d	1w	EVs improved kidney morphology and decreased fibrosis. However, EVs failed to preserve renal function.
UUO	Ji [28]	MSCs (human) umbilical cord	M (3)	IV	~ 2.5 mg* (10 mg/kg body mass)	UC	32-180 nm (NTA)	CD9, CD63, Alix. ,cyt C, calnexin (-)	SD rat (UUO), In vitro (rat TECs, mechanical stress)	M	6d	2w	EVs attenuated the progression of renal functional decline and improved fibrosis.
UUO	Shi [37]	MSC (rat) bone marrow	S	IV	~100 μg* (0.5 mg/kg body mass)	UC	50-600 nm (NTA)	CD9, CD63, HSP70	SD rat (UUO), In vitro (human TECs, TGF-β1)	M	1d	2w	Histology: EVs improved renal fibrosis. EVs reduced inflammation, oxidative stress, apoptosis, and fibrosis in TECs stimulated with TGF-β1
UUO	Yang [42]	EPCs (mouse)	M (3)	IV	~20 μg* (from 2x10^5 cells)	UC	100-1000 nm (TEM)	n.a.	C57BL/6 mouse, (UUO), In vitro (renal pericyte, TGF-β)	M	1d	5d	Histology: EVs improved EMT.
UUO	Choi [19]	MSCs (mouse) kidney	S	IV	2×107 EVs	UC	n.a.	n.a.	FVB/N mice (UUO), In vitro (HUVECs, TGF-β)	n.a.	1d	1w	Histology: EVs improved EMT, decreased PTC loss and inflammatory cell infiltration.
*Rodent HT*													
HT	Cambier [17]	CPCs (human)	M (5)	IV	350 μg	Conc- entation & MF	n.a.	*	C57BL/6J mouse (Ang II)	M	2w	4w	EVs decreased albuminuria, ameliorated renal structural injury and inflammation .
HT	Lindoso [34]	MSCs (human) adipose tissue	M (8)	IV	~20 μg (1.5x10^9 EVs)	UC	96-132 nm (NTA)	CD63, CD81	Wistar rat (DOCA-salt + UNX)	M	**1w**	8w	EVs improved renal function and ameliorated kidney damage by affecting kidney inflammation and EMT.
HT	Zou [47]	STC-like cells (pig)	S	IR	30 μg	UC	20-310 nm (NTA)	CD9, CD29, CD81, CD24	129-S1 mouse (RAS), In vitro (human TECs, AMA)	M	14	?	EVs improved renal function, alleviated tubular injury and restored mitochondrial function.
*Porcine*													
HT	Eirin [23]	MSCs (pig) adipose tissue	S	IR	~100 μg (1x10^10 EVs)	UC	20-400 nm (NTA)	CD9, CD40, CD81	Domestic pig (obese diet + RAS)	F	6w	10w	EVs improved renal function, decreased inflammation, improved medullary oxygenation and kidney fibrosis.
HT	Eirin [24]	MSCs (pig), adipose lean-EVs	S	IR	~100 μg (1x10^10 EVs)	UC	*	CD63, CD9, CD29, Beta1, MHCI, CD44	Domestic pig (obese diet + RAS) In vitro (human ECs)	F	6w	9w	Lean-EVs improved GFR and RBF, and increased tube number and length, and EC migration.
HT	Eirin [24]	MSCs (pig), adipose MetS-EVs	S	IR	~100 μg (1x10^10 EVs)	UC	*	CD63, CD9, CD29, Beta1, MHCI, CD44	Domestic pig (obese diet + RAS) In vitro (human ECs)	F	6w	9w	The above effects were decreased for MetS-EVs. MetS-EVs failed to restore renal angiogenic factors, improve microvascular density, or fibrosis.
HT	Song [14]	MSCs (pig), adipose tissue lean-EVs	S	IR	~100 μg (1x10^10 EVs)	UC	*	CD9, CD81, MSC markers	Domestic pig (obese diet + RAS), In vitro (activated T-cells)	F	6w	10w	Lean-EVs preserved GFR and improved kidney morhology by activating intrarenal regulatory T cells
HT	Song [14]	MSCs (pig), adipose tissue MetS-EVs	S	IR	~100 μg (1x10^10 EVs)	UC	*	CD9, CD81, MSC-markers	Domestic pig (obese diet + RAS), In vitro (activated T-cells)	F	6w	10w	The protective effects were decreased for MetS-EVs.
HT	Zhao [45]	MSCs (pig) adipose tissue	S	IR	1 x 10^11 EVs	UC	100-200 nm (NTA)	CD9, CD29, CD69	Domestic pig (obese diet + RAS)	F	6w	10w	EVs improved GFR and RBF and reduced fibrosis. Compared with MSC, EVs more significantly upregulated growth factor expression and decreased necroptosis.
DN, T1D	Duan [20]	SCs (human) urine	M (12)	IV	100 μg	UC	30–120 nm (TEM)	CD63, TSG101, HSP90B1 calnexin**	SD rat (Streptozocin), In vitro (human podocyte, glucose)	M	3d	12w	EVs preserved renal function and ameliorated glomerular damage.
DN, T1D	Jiang [29]	SCs (human), urine	M (12)	IV	100 μg	UC-DG	50–100 nm (TRPS)	CD63, CD9, CD8, MSC-EV markers	SD rat, (Streptozocin), In vitro (human podocyte, glucose)	M	3d	12w	EVs ameliorated glomerular damage but failed to preserve renal function.
DN, T1D	Grange [25]	HLSCs (human)	M (5)	IV	~100 μg (1x10^10 EVs)	UC	44-280 nm (NTA)	*	NOD/SCID/iL2Ry KO NGS mouse (Streptozocin)	M	5w	7w	EVs partially improved SCr, BUN, and histological tubular and glomerular damage and improved fibrosis gene expression.
DN, T1D	Grange [25]	MSCs (human), bone marrow	M (5)	IV	~100 μg (1x10^10 EVs)	UC	44-280 nm (NTA)	*	NOD/SCID/iL2Ry KO NGS mouse (Streptozocin)	M	5w	7w	EVs partially improved SCr, BUN, and histological tubular and glomerular damage and improved fibrosis gene expression.
DN, T1D	Ebrahim [22]	MSCs (rat), bone marrow	M (56), M (2)	IV	~30 ug* (100 μg/kg body mass)	UC	40–100 nm (TEM)	CD81, CD63	Allbino rat** (S**treptozocin)	M	8w 4d	12w 4d	EVs markedly improved kidney function, and reduced histological tubular and glomerular damage and improved kidney fibrosis.
DN, T1D with HU	Zhong [46]	MSCs (human), umbilical cord	M (4)	IV	~30 ug* (1.5 mg/kg body mass)	UC	30-500 nm (DLS)	n.a. (MSC-markers)	Balb/C mouse, (Streptozocin) with high UA In vitro (human PTCs)	M	2w	6w	EVs markedly improved kidney function, and reduced EMT.
DN, T1D with HU	Zhong [46]	MSCs (human), umbilical cord	M (6)	IV	~30 ug* (1.5 mg/kg body mass)	UC	30-500 nm (DLS)	n.a. (MSC-markers)	Balb/C mouse, (Streptozocin) with high UA In vitro (human PTCs)	M	2w	8w	EVs markedly improved kidney function, and reduced EMT.
DN, T1D with HU	Zhong [46]	MSCs (human), umbilical cord	M (8)	IV	~30 ug* (1.5 mg/kg body mass)	UC	30-500 nm (DLS)	n.a. (MSC-markers)	Balb/C mouse, (Streptozocin) with high UA In vitro (human PTCs)	M	2w	10w	EVs markedly improved kidney function, and reduced EMT.
DN, T2D	Duan [21]	MSCs (mouse), adipose tissue	M (12)	IV	100 μg	UC	30-150 nm (DLS)	CD9, CD63, CD81, TSG101	C57BL/KsJ db/db mouse, In vitro (mouse podocyte, glucose)	M	13w	25w	EVs decreased kidney structural damage and improved kidney function.
DN, T2D	Jin [30]	MSCs (mouse), adipose tissue	M (12)	IV	n.a.	Immun-P	60-500 nm (NTA)	CD9, CD63, CD81	C57BL/KsJ db/db mouse, In vitro (mouse podocyte, glucose)	M	13w	25w	EVs improved kidney function and reduced kidney structural damage.
DN	Nagaishi [35]	MSCs (rat), bone marrow	S	intra-renal	5.3 × 10^7 EVs (CD9 ELISA)	UC	40–100 nm (TEM)	HSP70, CD9, CD63	Rat (Streptozocin), In vitro (rat TECs)	n.a.	4w	5 or 6w	EVs ameliorated renal histological damage induced by DM and decreased immune cell infiltrates.
DN	Jin [31]	MSCs (mouse), adipose tissue	n.a.	n.a.	n.a.	Immuno-P	<100 nm (TEM)	CD9, CD63, CD81	In vitro (mice podocyte, glucose)	-	-	-	EVs protect against high glucose-induced EMT progression of podocytes.
(Other)Nx	He [26]	MSCs (mouse), bone marrow	M (3)	IV	30 μg	UC	~ 100 nm (TEM)	n.a.	C57BL6/J mouse (5/6 Nx)	n.a.	2d	1w 4d	EVs preserved renal function, decreased renal fibrosis, inflammation, and tubular damage.
(Other)Nx	Van Koppen [11]	MSCs (human), embryonic	M (4)	IV	7 μg	UC-DG	n.a.	n.a.	Lewis rat (5/6 Nx + L-NNA + salt), In vitro (human endothelial cell)	M	6w	11w	EVs had no effect on kidney function and histology. EVs improved angiogenesis in vitro.
(Other)GN	Cantaluppi [18]	EPCs (human), blood	S	IV	~60 μg* (30 μg/100g body mass)	UC	60-130 nm (NTA)	CD55, CD59	Wistar rat (anti-Thy1.1 Ab), In vitro (rat mesangial cells, anti-Thy1.1 Ab)	F	2d	2w	EVs preserved renal function and morphology and decreased inflammatory cell infiltration.
(Other) Toxic	Kholia [32]	HLSCs	M (4)	IV	100 μg* (1x10^10 EVs)	UC-DG	40-100 nm (TEM)	CD81, CD9, TSG101, CD81, CD107	NGS mouse (Aristolochid acid), In vitro (mouse TECs, AA).	M	3d	4w	EVs preserved renal function and prevented kidney histological damage and decreased immune infiltrates in the kidney.
(Other) Toxic	Kholia [33]	MSCs (human), bone marrow	M (4)	IV	100 μg* (1x10^10 EVs)	UC	80-500 nm (NTA)	CD9, CD63, CD81, GM130 (-)	NGS mouse (Aristolochid acid), In vitro (mouse TECs, AA).	M	3d	4w	EVs preserved renal function, prevented kidney histological damage, and decreased inflammatory infiltrates in the kidney.
(Other) Toxic	Ramirez-Bajo [12]	MSCs (mouse), bone marrow	M (2)	IV	100 μg	UC	22-370 nm (NTA)	CD63, CD9, MSC markers	C57BL/6 mouse (CyA)	M	**1d**	4w	EVs slightly improved BUN, did not improved morphology and failed to improve body weight.
(Other) Toxic	Ramirez-Bajo [12]	MSCs (mouse), bone marrow	M (2)	IV	100 μg	UC	22-370 nm (NTA)	CD9, CD63, MSC-markers	C57BL/6 mouse (CyA)	M	**2w**	4w	EVs reduced BUN slightly better than preventive intervention. Also, curative EVs decreased cyst formation and improved body weight.
(Other) Toxic	Zhang [44]	MSCs (human), umbilical cord	M (2)	IV	100 μg	UC	300-500 nm (EM)	MSC-markers	SD rat (CyA), In vitro (human TECs, CyA)	M	1w	4w	EVs improved renal function and ameliorated renal structural injury and oxidative stress.
(Other) Alport	Sedrakyan [36]	AFSC (mouse)	S	IC	200 ug	UC	50-500 nm (NTA)	CD9, CD63 , CD24, MSC-markers	Alport-TektdT mouse, In vitro (mice GEC)	n.d.	8w	36w	EVs improved renal function and decreased GEC damage.

Cell of EV origin: *AFSC* amniotic fluid stem cell, *CPC* cardiac progenitor cell, *EKC* embryonic kidney cell, *EPC* endothelial progenitor cell, *HLSC* liver stem cell, *MSC* mesenchymal stem cell, *SC* satellite cell, *STC* STC-like cell, *uSC* urine mesenchymal stem cell. Methods of EV measurement: *DLS* dynamic light scattering, *TEM* transmission Electron Microscopy, *NTA* nanoparticle tracking analysis, *TRPS* tunable resistive pulse sensing. EV markers: *CD* cluster of Differentiation, *HLA* human leukocyte antigen, *TSG101* tumor Susceptibility 101. CKD model: *HT* hypertension, *DM* diabetes, *RAS* renal artery steatosis, *UUO* unilateral ureteral obstruction, *Nx* nephrectomy, *AMA* Antimycin-A, *CyA* cyclosporine A, *PTC* peritubular capillary, *TEC* tubular epithelial cell, *TGF-β1* transforming growth factor β, *EMT* epithelial to mesenchymal transition. Route of EV administration: *IV* intravenous, *IC* intra-cardiac, *IR* intra-renal

~data regarding single dosage were extrapolated for illustrative purposes, where dosage was recalculated based on μg per body mass or 100 μg protein was approximated as 1*10^10 EV particles or EVs from 1x10^6 SCs

^*^asterisk marks immunodeficient mice strain

^#^EA indicates start of EV administration (days since UUO procedure, or streptozocin injection for diabetes models, or RAS for hypertension). End indicates study end (days/weeks after induction)

Studies in the UUO model (except one study [42] on EPCs) tested MSC-derived EVs, have utilized single therapeutic doses (ranging from 30 µg to milligrams of EV protein per animal), that were administered (intravenously) briefly (1 or 2days) after model induction (preventive treatments). One study (Chen et al. 2019) was performed in immune-deficient NGS mice and administered human adipose tissue MSC-derived EVs [10]. The study by Wang [38] has compared therapeutic effect between preventive administration of smaller doses of bone marrow MCSC-EVs isolated from young adult rats versus administration of EVs isolated from older rats. Only one study [28] has administrated 3, larger doses of EVs (single dose of approximately 2 mg protein per animal), starting from the 6th day after UUO surgery. Importantly, the studies in UUO varied regarding study quality and adherence to ISEV guidelines (Table 1 and Supplementary Table 1).

In contrast to the UUO model, studies in diabetes have utilized multiple doses of EVs that were mainly administered to animals with kidney disease (curative treatment). There were many different (multiple) doses of SCs-EVs, which are summarized in Table 1. Two studies in type 1 diabetes [20, 29] have administered urine SC-derived EVs (12 doses) briefly (3 days) after streptozocin injection (preventive treatment). The study by Grange et al. have administered curative treatments (5 doses of EVs) derived from either human bone marrow MSCs or HLSCs to immunodeficient NGS (NOD/SCID) streptozocin-induced mice. Another study in type 1 diabetes model (Ebrachim et al., 2018) has injected different (curative) doses of MSC-EVs into Albino rats. Also, one study (Zhong et al., 2019) has administered various doses of umbilical cord MSC-EVs (4, 6, or 8 doses) to a preselected subset of Streptozocin-induced type 1 diabetes mice with high uric acid concentrations [46]. Two studies were performed in type 2 diabetes (db/db) mouse model [21, 30] and injected multiple doses of adipose tissue MSC-derived EVs (12 doses, administered weakly), since the 13th week of age. Additionally, one study in diabetes [35] has utilized intra-renal MSC-derived EV injection of smaller EV doses, and documented histological findings.

Studies in hypertension were conducted in either porcine or rodent CKD models. Studies in porcine CKD (model of metabolic syndrome and renal artery stenosis) utilized single doses of adipose tissue MSC-EVs that were injected 6 weeks after RAS into the renal artery [14, 23, 24, 45]. Two of these studies have utilized MSC-EVs obtained from pigs with metabolic syndrome and from lean pigs [14, 23]. Conversely, two studies in rodent models of HT tested venous injection multiple EV doses. The first study, by Cambier et al., administered 5 doses (350 µg each) of smaller EVs derived from human CPCs, in a mouse model of cardiac hypertrophy and kidney injury. These EVs were administered retro-orbitally, after 2 weeks of chronic infusion of Angiotensin II. The second study, by Lindoso et al., injected 8 smaller doses of human adipose tissue MSC-derived EVs, 2 weeks after nephrectomy (1 week after administration of DOCA-salt). The third study, by Zou [48] injected single doses of porcine STC-like cell-derived EVs into the stenotic kidneys of RAS mice.

All studies in toxic CKD were performed in rodent models and utilized multiple EVs doses. The two studies (by Kholia et al.) were performed in an immunodeficient NGS mouse model, that was induced by injection of aristolochic acid. In these studies, human HLSC or human bone marrow MSC-derived EVs were injected intravenously, with the first EV dose administered briefly (3 days) after toxicant injection. Another study by Ramirez-Bajo utilized either preventive (the first EV-treatment dose administered 1 day after cyclosporine A administration) or curative character of mouse bone marrow MSC-EV administration (first EV-treatment dose administered 2 weeks after cyclosporine A administration). Another study by Zhang et al. [44] administered human umbilical cord MSC-derived EVs one week after administration of cyclosporine A.

Only two studies were performed in 5/6 Nephrectomy and both of these studies tested smaller EV doses. One study He et al. [26] injected 3 doses (30 µg dose) of bone marrow EV-based treatment (preventive treatment). The second study [11] utilized multiple injections of small doses of human embryonic EVs into rats with late CKD stage.

Finally, the studies that focused on genetically modified EVs overexpressing defined miRNAs (or silencing experiments) have been presented separately in Supplementary Table 2.

### Quality of Extracellular Vesicle-Research

The International Society for Extracellular Vesicles had proposed a set of standards to characterise EVs preparations for preclinical studies. They include adherence to EV extraction and characterisation protocols, appearance in transmission electron microscopy (TEM), paired with counting of EVs with particle enumeration methods, expression of the “EV-enriched” markers, or the absence of expression of non-EV components [48]. In line with those guidelines, we assessed the quality of the included studies by investigating the EV-specific experiments. Differential ultracentrifugation (UC) was the most commonly used EV separation technique, while other methods, such as density gradient-UC [12, 32], polymer-based precipitation [21, 40], and immunoaffinity capture [30, 31], were used by 5–15% of studies. To improve the specificity of EV separation, 7 studies had utilised complementary techniques following the primary step, such as ultrafiltration, or application of density gradients [13, 17, 20, 25, 29, 30, 33, 41].

The studies documented that the morphology of the preparations was consistent with the characteristics of EVs by providing vesicle characteristics (most commonly TEM and NTA results), and by measuring the EV enriched markers. In case of several studies, the ISEV criteria have been met in their previous publications. Together, most authors (60%) provided characterization of vesicles with two complementary techniques. Positive EV markers were measured by 80% of the authors; however, these markers were mostly limited to transmembrane proteins. Also, the number of the reported markers varied between studies. Two studies have supplemented those findings with negative markers to exclude the presence of larger types of EV. Importantly, while reviewing the studies it was visible that although in the early phase of EV research in CKD, many authors did not provide EV characteristics, the adherence to the ISEV standards is improving gradually with time, which is in the right direction - towards further standardisation of the results, and enabling more precise comparison between the studies.

In a formal bias analysis, we investigated the publication risk of bias using the selected items from the CAMARADES checklist [49]. The included studies were published in impacted journals, provided statements regarding compliance with regulatory requirements, and conflict of interest. Randomisation of animals was reported in a half of all studies. Measurements and analysis of histology outcomes was only performed blindly by the authors in one third of the included studies. All the results of the quality assessment at individual study level are summarised in Supplementary Table 1.

### Therapeutic Effect of Extracellular Vehicle-Based Therapy on the Kidney Function Decline

Of all eligible publications examining EV-based therapy in CKD animal, more than 90% of studies reported significantly improved kidney histology, and more than 80% of eligible studies confined significantly improved markers of kidney function (Table 1). Also, some publications observed significant protective effect on renal structure, although no statistically significant changes in ether creatinine or eGFR/urea were detected [11, 29], or observed inconsistent or borderline changes [13, 38]. Only one study in nephrecomized rats reported the lack of protective effect on either kidney function or the structural measurements [12]. However, this particular study has utilised smaller EV doses.

There were 26 studies eligible for meta-analysis of renal decline outcomes: 19 studies (total 27 comparisons) that measured plasma creatinine concentrations, 15 studies (total 25 comparisons) that measured plasma urea concentrations, and 7 studies (total 9 comparisons) that measured glomerular filtration rate (GFR) (See Fig. 1).

As main approach to evaluate the combined EV therapeutic effect (on plasma creatinine and urea), we have performed the meta-analyses for all records, excluding the studies that did not report any EV characteristics (EV-enriched markers, or EV size/visualisation with TEM), and studies performed in large animal (porcine) models (see Fig. 3). As additional approach, we have performed sensitivity analyses for all the eligible studies, to evaluate the impact of these exclusions on the overall findings (Supplementary Fig. 3). The study design and time-points for the individual study cohorts within creatinine and urea analyses are characterised in Fig. 3 and Supplementary Fig. 3. The GFR analysis was mostly done in hypertension and contained studies that reported creatinine clearance (3 studies, all in rats) and studies with a multi-detector computed tomography (4 studies, all in pigs).

The treatment of animals with EVs has consistently improved (*p*-values < 0.001 for all 3 outcomes) all three parameters, supporting the potential protective properties in CKD (Fig. 3, and Supplementary Figs. 1-3). The graphical illustration of the treatment effect of EV-based therapy on plasma creatinine and plasma urea based on CKD model is shown in Fig. 3. The SMDs for both markers were considerably different between CKD models (Fig. 3A and B). For plasma creatinine the SMDs were: -2.06 (95% CI: -4.46; 0.35) for UUO, -2.97 (95% CI: -5.31; -0.63) for hypertension (HT)-induced CKD, -8.97 (95% CI: -16.41; -1.53) for diabetes-induced CKD, -1.65 (95% CI: -2.42; -0.86) for toxin-induced CKD, and -2.04 (95% CI: -3.23; -0.85) for 5/6 nephrectomy (Fig. 3A). For plasma urea the SMDs were -3.06 (95% CI: -6.02; -0.10) for UUO, -4.52 (95% CI: -7.73; -1.32) for diabetes-induced CKD, -1.25 (95% CI: -2.24; -0.25) for toxic CKD, and -0.63 (95% CI: -1.61; 0.35) for 5/6 nephrectomy (Fig. 3b). The subsequent analysis including all study cohorts that reported renal decline is illustrated in Supplementary Fig. 3.

The analysed outcomes showed significant asymmetry of the results in funnel plot analyses indicating a possibility of potential bias. In addition, the results of the Egger regression-based test (*p-values* < 0.001 for all 3 outcomes) supported the existence of small publication bias. The trim and fill procedure identified 4 missing studies for creatinine, and 1 missing study for urea, however did not influence the overall findings for either outcome measures. Statistical heterogeneity was high in all analyses (with I-squared index exceeding 75%), and we have conducted a stratified meta-analysis to investigate potential sources of heterogeneity in creatinine and urea concentrations.

The stratified analyses for plasma creatinine indicated that timing of the therapy (curative vs preventive), and administered dose of the EVs were potential determinants of the therapy efficacy (Supplementary Fig. 2A). Considerably higher treatment effect was observed in diabetic (-8.97 (95% CI: -16.41; -1.53)) than in non-diabetic (-2.24 (95% CI: -3.10; -1.38)) models of CKD (p-value for the difference = 0.06). The treatment effect was -6.72 (95% CI: -11.77; -1.67) in animals receiving multiple doses of EVs in comparison to -2.21 (95% CI: -3.60; -0.81) in animals receiving single dose of EVs (p-value for the difference = 0.09). The treatment effect was -8.68 (95% CI: -15.19; -2.17) in animals receiving curative treatment in comparison to -1.56 (95% CI: -2.28; -0.85) for animals receiving preventive treatment (p-value for the difference = 0.03).

The directions of the observed differences in urea concentration between subgroups of diabetes-induced CKD versus other CKD models were similar to the pattern observed for creatinine concentrations, albeit not significant (SMD -4.52 (95% CI: -7.73; -1.32) vs SMD -1.97 (95% CI: -3.40; -0.54); p-value for the difference = 0.15). Also, there was a trend towards better treatment response in urea for multiple EV doses ((-3.68 (-5.87; -1.48) vs -1.65 (-3.26, -0.04); p-value for the difference = 0.1)), and later time-point of EV administration ((-5.00 (95% CI: -8.43; -1.56) vs -1.82 (95% CI: -3.27; -0.37); p-value for the difference = 0.09). However, the number of included studies was relatively low and CIs were wide, indicating low statistical power to detect significant differences. The SMDs in plasma urea were -3.42 (95% CI: -5.64; -1.19) in mice, and -3.00 (95% CI: -6.32; 0.35) in rats (p-value for the difference = 0.83). There were no significant differences in creatinine or urea reduction by EV source (xenogeneic vs. allogenic); however, statistical power was also low.

To evaluate the underlying differences in the effectiveness of EV-treatment we analysed their biological effect across the included studies. We assessed the histological findings, biochemical data, as well as active molecules contained within EVs, and their involvement in CKD pathways. In order to illustrate the biological implications of vesicular miRNAs in the kidney we performed interaction network analysis between the target genes of the miRNAs identified based on the literature search. In the following section, we catalogue these findings according to the mechanism underlying the EV protective effect and the CKD model.

### Mechanisms of Action of Extracellular Vesicles and Enclosed Molecules According to the Chronic Kidney Disease Model

A primary component, that is common to all CKD aetiologies, is the progressive fibrosis of kidney cells leading to impaired renal function. Thus, one of the main features to consider when assessing the EV nephroprotective capacity involved modulating fibrosis-related mechanisms (decreasing inflammation, decreasing cell damage and inducing angiogenesis, improving anti-ROS response), and treating fibrotic process by regulating the expression of pro-fibrotic programs of kidney cells. To induce the anti-fibrotic protective response, EVs were derived either from stem cells (SCs), kidney cells, or were generated from engineered cells, by using lentiviral RNA transfer. The cell types that were utilised as EV source, based on their involvement in CKD mechanism, are summarised in Supplementary Table 3 and represented in Fig. 4.

Twenty-five of the eligible studies showed that EVs ameliorated kidney histology: 13 studies reported reduced glomerulosclerosis (GS) and 23 studies reported reduced tubulointerstitial fibrosis (IF). Also, 26 studies supplemented the histological findings with the decreased expression of pro-fibrotic mediators (Supplementary Table 2). In the early studies in experimental CKD, He et al. investigated nephroprotective properties of MSC-EVs in animal models of Nx and UUO, showing ameliorated functional damage and reduced fibrosis. [26, 27]. MSCs-EVs were as effective as MSCs themselves in accelerating functional recovery from CKD in UUO mice in vivo [27]. These EVs inhibited TGF-β1-induced morphological changes in mice tubular epithelial cells in vitro, and these effects were attributed to their carried miRNA cargo [27]. Similarly, Choi et al. reported that MSC-EVs reversed morphological changes induced by TGF-β1, ameliorated peritubular capillary rarefaction, and reduced IF in UUO mice [19]. In line with these findings, MSC-EVs were subsequently reported to reduce GS and/or TF in the settings of UUO [11, 28, 39, 41], hypertension [15, 23, 24, 45], and diabetic CKD [22, 25, 35]; where the damage was improved via mechanisms involving RNA or protein transfer. Among the studies that attributed the protective properties of MSC-EVs to their miRNA content, 3 studies performed microarray profiling inside MSC-EVs - the predicted targets of the vesicular miRNAs were implicated in extra-cellular matrix and collagen synthesis and degradation [25, 27, 39]. Further, Zhong et al. demonstrated that EVs ameliorated IF in STZ-induced diabetic mice [46] via modulation of the kidney expression of P15 and P19 molecules (leading to an improved cell cycle arrest) by EV-enclosed miR-451a. Jin et al. found that coincubation of MSC-EVs with podocytes treated with glucose decreased EMT progression via regulation of ZEB2 transcription factor through EV-encapsulated miR-215-5p [31]. Three studies in animal UUO administered engineered EVs that over-express protective miRNAs capable of an anti-fibrosis effect. First, the study by Wang reported that the addition of let7c-MSC‐EVs repressed collagen type IVα1, α-SMA, and TGF-βR1 expression in rat TECs that had been exposed to TGF-β1 [40]. Next, miR-26a-HEK-derived EVs were reported to suppress the TGF-β signalling pathway in the injured kidney by targeting TGF-β1 and CTGF [43]. Third study by Wang et al showed that intramuscular injection of miR-29-EVs into UUO mice reduced TGF-β3, and decreased interstitial collagen accumulation in the kidney [41], suggesting that EV anti-fibrotic protective effect was exerted through the TGF-β singling pathway inhibition. Among studies that attributed the protective anti-fibrotic properties of MSC-EVs to their protein content, the study by Ji et al. performed global EV proteome profiling [28] identifying enrichment of the TGF-β1, TLR, VEGF, and ubiquitin-related enzymes - CK1δ and βTRCP. Administration of these MSC‐EVs to UUO rats improved fibrosis by regulating Yes-associated protein (YAP), a co-activator of the Hippo pathway. By contrast, the EV protective effect was impaired with EV-CK1δ and β-TRCP knock-down.

Stem cell-EVs were also documented to modulate renal fibrosis through their anti-inflammatory and immunomodulatory effect. The anti-inflammatory effect of EVs was well-assessed in hypertension (HT) and the metabolic-related CKD models. Regarding HT, the immunomodulatory EV effect was attributed to a reduced renal immune infiltration and modulation of functional programming of macrophage populations. Eirin et al. showed that intra-renal administration of MSC-EVs containing IL-10 mRNA attenuated kidney inflammation in the MetS/RAS pigs by switching renal macrophages from M1 to M2 phenotype [23]. Consistent with this macrophage reprogramming mechanism, multiple studies showed that EVs reduce the expression of pro-inflammatory mediators (such as TNF-α, MCP-1, IL-6, and iNOS) and increase the expression of anti-inflammatory mediators (such as IL-10) in the kidney [13, 17, 21, 24, 25, 33, 37] or circulation [15, 17, 24, 45]. Eirin et al demonstrated that the protective effect was diminished when MSC-EVs derived from pigs with metabolic syndrome were used as compared with MSC-EVs from lean pigs [24]. Extending the mode of action of lean MSC-EVs, Song et al. reported that induction of intrarenal regulatory T cells through TGF-β was required for their anti-inflammatory effects [15]. Further extending the mechanisms of MSC-EVs in HT, Lindoso et al. reported down-regulation of EMT by modulating the kidney miRNA signature as well as induction of immunomodulatory mechanisms in EV-treated DOCA-salt rats [34]. EVs administration has led to normalised renal function, blood pressure, inhibited macrophage recruitment, down-regulated the kidney expression of pro-inflammatory mediators (PAI1, MCP-1, IL-6), and decreased the markers of tubular and glomerular injury. Zhao et al. compared the effects of the administration of MSCs and MSCs-EVs into the stenotic kidney [45]. Both therapies demonstrated beneficial effects on fibrosis, inflammation, and microvasculature. However, in this setting, EVs increased the expression of growth factors more effectively than MSCs. This was explained by EV enrichment with miR-532-5p, which can modulate Angiopoietin-1. Finally, Cambier et al. investigated CPC-EVs and YF1 RNA fragment, and reported improved cardiac hypertrophy, kidney function, as well as reduced fibrosis, and diminished inflammation through induced secretion of IL-10 in plasma, heart and kidney [17].

As outlined above, EVs were shown to mediate their their anti-inflammatory nephroprotective effect through interactions with various types cells of innate and adaptive immune system, including macrophages, monocytes and T-cells. Consequently, their anti-inflammatory role might be modulated differently between immunocompetent animals compared with the immunodeficient animals, that lack adaptive immune response. Immunodeficient animal strains (such as NGS, Balb/C, nude) were used by several studies utilizing human EV transplant, in order to avoid xenogeneic EV transplant-induced immune response and toxicity. Two of those studies that were conducted in toxic CKD have performed immunostaining in kidney tissue of NGS mice, to report that the animals receiving EV-based therapy had a significantly lower expression of CD45 positive cells, FSP-1 and α-SMA positive myofibroblasts. As these mice lack an adaptive immune system due to their genetic background, the authors concluded that the CD45 (leucocyte common antigen) positive cells were likely to be part of the innate immune system, suggesting that inflammatory responses can be modulated independently of adaptive immunity.

Regarding the diabetic CKD, Grange et al. reported the protective effect of MSC-EVs and HLSC-EVs on structural damage, glomerulosclerosis, and tubulointerstitial fibrosis in a mouse model of diabetes induced by streptozotocin (STZ) [25]. Utilising RNA profiling of the kidney tissue the authors found that SC-EV treatment normalised a significant number of the fibrosis-related and inflammation-related genes that were induced by diabetes, including MMPs, TIMPs, and certain chemokines. Along with changes in renal morphology and the patterns in gene expression, an in silico analyses of the EV-enclosed miRNAs, identified putative EV targets belonging to pro-fibrotic signalling. Likewise, Nagaishi et al. reported the anti-inflammatory, and cell protective effect of MSCs in diabetes, which was modulated through EVs [35]. Those MSC-EVs protected against apoptosis, ameliorated TECs damage, decreased inflammatory infiltrates, and reduced fibrous component of the interstitial space.

Multiple studies investigated EV effect focusing on kidney cell damage/survival pathways, including apoptosis [20, 21, 29, 30, 47], necropoptosis [45], and autophagy [22, 30]. Regarding diabetes-induced CKD, Duan et al. showed that SC-EVs promoted podocyte survival in studies utilising rodent models of T1D and T2D [20, 21]. In the first study, injection of urinary SC-EVs into STZ-induced rats improved renal function decline, glomerular structure, decreased glomerular damage markers, and reduced the kidney expression of TGF-β1 [21]. Secretion of miR-16-5p from uSC-EVs ameliorated glucose-induced podocyte injury by targeting VEGF signalling, whereas administration of miR‐16‐5p-uSCs led to a decrease in the levels of kidney VEGFA, TGF-β1, and pro-inflammatory mediators (MCP-1, TNF-α), which were increased following the establishment of DN. In the second study by this group, the addition of MSC-EVs containing miR-26a-5p into human podocytes inhibited TLR4 and NF-κB/VEGFA signalling induced by glucose [20]. In line with this, the injection of miR-26a-5p-EVs decreased glomerular structural abnormalities in db/db mice. In another study in db/db mice, MSC-EVs ameliorated renal functional decline, improved podocyte damage, and inhibited apoptosis. [30]. These effect were mediated through miR-486 and inducing autophagy flux through modulation of the Smad1/mTOR signalling pathway. Further, SC-EVs express miRNAs involved in vasculature protection and secrete growth factors, which decrease endothelial cell (EC) damage. Chen et al. showed that the delivery of GNDF from MSC-EVs activated the kidney SIRT1/eNOS pathway, resulting in significantly decreased IF in UUO mice [11]. Those GNDF-MSC-EVs decreased apoptosis, and induced angiogenic activities in vitro. Further, EPSs were documented to reduce glomerular mesangial, and endothelial cell injury and to enhance microvascular repair in AKI and CKD [18]. Finally, AFSC-EVs were shown to mediate anti-apoptotic, and pro-angiogenic effects in mice with Alport Syndrome [36].

The anti-oxidant response was also involved in affording protection following EVs administration in CKD [37, 44, 47]. However, the involved mediators were not investigated, except for one study by Zou et al. [47]. This study demonstrated that kidney ST-like cell-EVs transfer mitochondria, which remain functional in the recipient TECs. Administration of these EVs improved oxidative stress, decreased cell damage, and improved kidney perfusion.

The graphical representation of the current concept of EV-enclosed active molecules based on their downstream mechanism in the kidney is shown in Fig. 4, and the detailed characteristic of study design of the experiments evaluating particular EV effector molecules is summarised in Table 2. Also, the results of our in silico analysis showing (A) the overlap in the expression patterns of the protective miRNAs in EVs from different cell sources (mesenchymal stem cells, endothelial cells, and urine, and (B) target-target interaction network among miRNA targets with underling KEGG ontologies and GO terms, are shown on Fig. 5.

**Table 2:** Details of the experimental design of studies evaluating EV effector molecules. Study design, EV characteristic, target and main study findings are outlined

CKD model	Study	Cell	AD	CM	EV size	EV markers	Animal model	Effector	Target	Main findings
UUO	Wang [38]	MSCs (r):	IV	Column	n.a.	MSC-EV markers	Fisher 344 rat (UUO), In vitro (h TECs, TGF-β1)	miR-294, miR-133, (mimic/ inhibitor)	TGF-β1	Old rat miRNA-depleted EVs failed to improve kidney fibrosis. Injection of mimic miRNAs reversed those effects. Also, overexpression of miRNAs mitigated TGF-β1-mediated EMT in TECs..
UUO	Chen [10]	GDNF--MSCs (h)	IV	UC	30-150 nm	CD9, CD63, CD81	Nude mice* (UUO), In vitro (human EC)	GDNF (GDNF-EVs)	SIRT1/eNOS	GDNF-MSC-EVs were more effective in reducing fibrosis than GFP-MSC-EVs. However, GDNF-MSC-EVs did not improve renal function.
UUO	Ji [28]	MSCs (h)	IV	UC	30-180 nm	CD9, CD63, Alix	SD rat (UUO), In vitro (rat TECs, mechanical stress)	CK1δ, β-TRCP (KO-EVs: shCK1δ- and shβ-TRCP-EVs)	Yes-associated protein (YAP)	EVs overexpressed CK1δ and β-TRCP protein. Administration of EVs increased CK1δ, β-TRCP, and decreased expression of YAP in kidney tissue. CK1δ, and β-TRCP knockdown decreased anti-fibrosis effectiveness of EVs.
UUO	Shi [37]	MSC (r)-silenced	IV	UC	50-600 nm	CD9, CD63, HSP70	SD rat (UUO), In vitro (human TECs, TGF-β1)	MFG-E8 (MFGE8–silenced-EVs)	RhoA/ROCK pathway	Protective effects of EVs on kidney histology, fibrosis, and inflammation were abolished by the inhibition of MFG-E8 in EVs.
UUO	Wang [41]	miR-29-Satellite cells (m)	IM	UC	87-93 nm	TSG101	C57BL/6J mouse (UUO)	miR-29 (miR-29-EVs)	TGF-β3	Injection of miR29-EVs attenuated renal histology and fibrosis.
UUO	Zhang [43]	miR-26a-EKC (h)	IM	UC	50-300 nm	TSG101	C57BL/6J mouse (UUO)	miR-26a (miR-26a-EVs)	CTGF, TGF-β1	Injection of miR-26-EVs attenuated renal fibrosis by limiting CTGF.
UUO	Wang [40]	let-7C-MSCs (h)	-	PC	n.a.	?	In vitro (mouse TECs, TGF-β)	let-7c	TGF-β1	let7c-EVs inhibited TGF-β1 in vitro
HT	Cambier [17]	CPCs (h)	IV	MF	n.a.	*	C57BL/6J mouse (Ang II)	Y-RNA	IL10	YF1-EV-RNA improved kidney function, and diminished renal inflammation and fibrosis.
HT	Zou [47]	STC-like cells (p)	IR	UC	20-310 nm	CD9, CD29, CD81	129-S1 mouse (RAS), In vitro (human TECs, AMA)	mitochondia, mitochondial DNAs	-	STC-like cells-EV-mitochondria remained functional and acquired TEC function.
HT	Eirin [23]	MSCs (p) adipose tissue (IL-10 KD)	IR	UC	30-400 nm	CD9, CD40, CD81	Domestic pig (obese diet + RAS)	IL10 mRNA ( KO EVs)	-	Compared with MSC-EVs, the protective effects on kidney morpology, renal function, and macrophage phenotype were blunted for KO-EVs.
HT	Zhao [45]	MSCs (p), adipose tissue	IR	UC	100-200 nm	CD9, CD29, CD69	Domestic pig (obese diet + RAS)	miR-532-5p	Growth factors?	microRNA-532-5p expression was upregulated in stenotic kidneys, possibly by its delivery by EVs.
DN, T1D	Duan [20]	SCs (h) urine	IV	PC	30–120 nm	CD63, TSG101, HSP90B1 calnexin -	SD rat (Stz), In vitro (human podocytes, glu)	miR-16-5p (miR-16‐5p-EVs and KO EVs)	VEGF	Compared with uSCs‐EVs, the hyperplasia of mesangial matrix and kidney function were more alleviated for miR-16-5p-EVs.
DN, T1D	Zhong [46]	MSCs (h), umbilical cord	IV	PC	30-500 nm	MSC-EV markers	Balb/C mouse, (Stz) with hyperurycemia In vitro (human PTCs)	miR-451a	P15, P19	Expression of miR was enriched in EVs compared with MSCs. Injection of EV-miR-451a (agomir) ameliorated tubular damage, and reduced EMT by inhibiting CKIs.
DN, T2D	Duan [21]	MSCs (m), adipose tissue	IV	UC	30-150 nm	CD9, CD63, CD81, TSG101	C57BL/KsJ db/db mouse, In vitro (mouse podocyte, glu)	miR-26a-5p (miR-26a-5p-EVs, KO EVs, miR antagomir)	TLR4	Administration of EVs induced miR-26a-5p and decreased TLR4 expression in kidney tissue. KO of EVs failed to induce any improvement in kidney function or renal histology. Delivery of miR-26a -5p-EVs to podocythes reduced apoptosis.
DN, T2D	Jin [30]	MSCs (m), adipose tissue	-	Immuno-P	60-500 nm	CD9, CD63, CD81	In vitro (mouse podocyte, glu)	miR-486 (KO EVs)	Smad1	miR-486 inhibition reduced the protective role of EVs in high glucose-induced podocyte damage.
DN, T2D	Jin [31]	MSCs (m), adipose tissue, miR-215-5p-KO	-	Immun-P	<100 nm	CD9, CD63, CD81	In vitro (mice podocyte, glu)	miR-215-5p (KO EVs)	ZEB2	KO EVs failed to modulate glucose-induced podocyte migration in vitro, while transfection with miR-215-5p mimics in podocytes reversed the effect. miR-215-5p (mimic) blocked HG-induced ZEB2 expression in vitro.
(Other)GN	Cantaluppi [18]	EPCs (h), blood	IV	UC	60-130 nm	CD55, CD59	Wistar rat (anti-Thy1.1 Ab), In vitro (rat mesangial cells, anti-Thy1.1 Ab)	Factor H, CD55 and CD59 mRNAs, miR-126, miR-296	C5b-9	EVs reduced cell damage and death in the presence of rat or human sera. These effects were blunted when EVs were treated with RNAse.
(Other) Alport	Sedrakyan [36]	AFSC (m)	IC	UC	50-500 nm	CD9, CD63 , CD24	Alport-TektdT mouse, In vitro (mice GEC)	miRNAs, VEGFR1, sVEGR1 (KO EVs)	VEGF	KO EVs failed to modulate kidney VEGF expression.

Cell of EV origin: *AFSC* amniotic fluid stem cell, *CPC* cardiac progenitor cell, *EKC* embryonic kidney cell, *EPC* endothelial progenitor cell, *MSC* mesenchymal stem cell, *SC* satellite cell, *STC* STC-like cell, *uSC* urine stem cell. *AD* EV administration, *CM* EV concentration method, *UC* ultracentrifugation, *PC* precipitation, *MF* microfiltration, *Immuno-P* immuno-precypitation. Methods of EV measurement: *DLS* dynamic light scattering, *TEM* transmission Electron Microscopy, *NTA* nanoparticle tracking analysis, *TRPS* tunable resistive pulse sensing. EV markers: *CD* cluster of Differentiation, *HLA* human leukocyte antigen, *TSG101* tumor Susceptibility 101. CKD model: *HT* hypertension, *DM* diabetes, *RAS* renal artery steatosis, *UUO* unilateral ureteral obstruction, *Nx* nephrectomy, *AMA* Antimycin-A, *CyA* cyclosporine A, *PTC* peritubular capillary, *TEC* tubular epithelial cell, *TGF-β1* transforming growth factor β. Target *NOS endothelial nitric synthase*, *CTGF* Connective tissue growth factor, *IL10* Interleukin 10, *Smad 1* SMAD Family Member 1, *TLR4* Toll-like receptor 4, *EMT* epithelial to mesenchymal transition

## Discussion

Our qualitative review shows that the administration of EVs has consistently ameliorated functional, structural, and molecular measurements in studies regarding progressive kidney disease. The published data imply that SC-EVs mediate nephroprotection by influencing kidney fibrotic genes and exerting immunomodulatory and cell-protective activities. Additionally, our meta-analysis confirmed an improvement in renal function decline in CKD animals receiving EV-based treatment in comparison to untreated CKD controls. A major advantage of using EVs over stem cells themselves is to avoid the potential risks of tumorogenesis or maldifferentiation of the engrafted cells. EVs were postulated to represent a less immunogenic, and non-mutagenic to the recipient compared with other gene delivery vehicles [50]. Thus, we suggest EV-based regenerative approaches could offer a safer ‘off-shelf’ therapy for patients with CKD.

Knowing the particular type of study design and the factors influencing the efficacy of EV-based treatment may facilitate future experimental studies and may help design studies in specific patient populations. Thus, for the studies with available creatinine and urea data, we performed univariable stratified meta-analyses to investigate candidate predictors for EV-based therapy effectiveness, by CKD setting. In our findings, the differences in the functional efficacy of EV therapy appear to be model dependent. A considerable proportion of the animal records included in our analysis involved diabetic CKD models. Despite the current recommended multidisciplinary treatment including intensive glycemic control, tight blood pressure control, and renoprotective therapy such as renin-angiotensin-aldosteron inhibitors and sodium-glucose transport protein 2 inhibitors [51], there is still large residual risk of CKD progression in people with diabetes [52], indicating that additional CKD prevention options for people with diabetes are needed. The results of our meta-analysis show EV therapy is a promising approach for CKD progression in experimental diabetes and may be worthy to consider the possibility of clinical application. The calculated treatment effects were considerably weaker in other CKD aetiologies, which is in line with a results of a previous analysis by Papazova et al., who evaluated stem cell therapies in various pre-clinical models of CKD [16]. However, their study did not included treatment with EVs.

Secondly, we observe significant differences in the functional efficacy of EVs between the preventive and curative character of EV administration. Among the studies included, several (studies in UUO and two studies in type 1 diabetes), have utilized preventive EV administration. Their findings varied regarding the effect on renal decline outcomes, with a considerable proportion failing to document that administered EVs have significantly reduced plasma urea or plasma creatinine concentrations [38, 10, 29]. Moreover, the recent study performed in a rat model of toxin-induced CKD [12] has reported an increased effect of MSCs and MSC-EVs on kidney function decline/renal histology to be associated with later treatment administration, associating kidney inflammation as the main factor to renoprotective effects of these treatments. This is in agreement with the results from experimental studies that documented that the stem cell-EV uptake in the normal kidney tissue is very limited and could be specifically induced with a more severe tissue injury [19, 23, 28, 43, 53]. Several in vivo (or ex vivo) studies have been performed regarding EV biodistribution in AKI [53] and CKD-UUO animals [19, 28, 43], to identify that intravenously injected EVs were specifically retained by the damaged kidney and were engrafted only to a limited (20 times less) extent by the non-affected kidney [28]. Those EVs homed to TECs or PTCs, while fewer EVs were detected in the glomeruli (reviewed in 54). Moreover, animal studies in HT have shown that, following intra-renal administration, EVs engrafted specifically into the post-stenotic kidney, and that these EVs accumulated in TECs, and in kidney infiltrating macrophages [23]. Concurrently, the specificity of EVs towards homing in injured kidney was hypothesized to involve infiltrating leucocytes or increased surface markers on parenchymal kidney cells during inflammation [54]. However, the existence of complex types of communication between EVs and kidney cells including various receptors/ligands implicated has been suggested. As one mechanism to enter cells, EV express MSC surface adhesion molecules, such as tetraspanins (CD44, CD29). It has been shown that treatment with antibodies against those CD molecules prevented MSC-EVs from entering TECs [55, 56]. Conversely, recent data suggested that EVs rely on phosphatidylserine as target receptors, and characterized the T cell Ig and mucin domain–containing family molecules (TIMs) as receptors binding EVs [57]. On Tcells, EVs can directly interact with the T-cell membrane receptor Tim-4 [57, 59]. In the kidney, another TIM family molecule - Tim-1 (or kidney injury molecule -1) is specifically expressed in the damaged epithelium [58]. KIM-1 expression is undetectable in the normal kidney, while it specifically increases in TEC membranes during the early stage of AKI, conferring them phagocytic functions [60] while in progressive CKD, KIM-1 increases gradually with the disease stage, and it is involved in mediating the inflammatory response. This supports the notion that availability of kidney TIM-1 (and leucocyte TIM-4) may be another mediator of EV therapeutic properties [61, 62]. Moreover, conjugation of EVs with the KIM-1 targeting antibody has been shown to enhance the retention of exogenously administered EVs in the kidney and endowed with increased therapeutic anti-inflammatory properties in murine renal artery stenosis [63]. Nevertheless, the complete mechanisms behind the interaction between EVs and injured kidney cells remain poorly understood, are complex, and involve many mediating factors. Together, the gathered data imply the need for a better understanding of EV uptake, and the need for new approaches to enable specifically targeting retention, and uptake of EVs into the kidney cells [64]. We believe, though that future genetic and structural modifications of the isolated EVs will accelerate therapeutic application and will further improve the EV-based therapy efficacy.

Third, multiple EV-doses were considerably more effective in reducing renal decline. Accordingly, it would be worthy to model the relationship between the total EV dosage and the magnitude of the therapeutic effect. This task, however; requires complete, standardized dataset with regards to the dosing (f.ex the number of particles used (per body mass)), and as such we postulate this issue should be resolved by further studies. Phase 1 trials may be designed to address the safety of EV treatment, specifically focusing on EV pharmacokinetic profile, and establish the amount of EVs that causes the therapeutic effect without inducing toxicity.

Experimental studies have not only implicated stem cell-derived EVs in kidney protection but also pointed to the role of EV-enclosed miRNAs [65]. Since the miRNA depleted EVs do not present any protective properties in the kidney injury, it is likely that those miRNAs contribute to the kidney cell reprogramming [65]. Based on these findings, stem cell-derived miRNAs were identified as effectors of the EV nephroprotective benefits, implicated in inhibiting fibrosis and cell damage pathways in CKD setting. Nethertheless, many of the studies focused on isolated microRNA-gene (or gene-gene) relations and did not address the complexity of their interactions. As the protective effect of EVs may be a result of multiple miRNAs, a more comprehensive understanding of the global mechanisms and related EV secretome is needed. Additionally, the exact pathways involved in the immune-modulatory effects EVs in CKD may also need further investigation. In the stratified analyses, the therapeutic effect of EV-based treatment on renal decline was considerably stronger in diabetic than in non-diabetic animals. This differences in EV effectiveness might be due the underlying differences in study design, or may be attributed to anti-diabetic properties of EVs. Noteworthy, the treatment with MSC-EVs did not significantly affect insulin tolerance [15, 23, 24] or ameliorate hyperglycemia [21, 25, 29], indicating that inflammation or other mechanism altered by EVs may play role, at least in this particular setting.

The results of our analyses prompted us to highlight the relevant areas for future research. Several features, such as the CKD model, time of EV administration, and EV dose appeared to have considerable effects on therapy effectiveness in our univariable analyses. However, definitive conclusions about independent predictors of EVs efficacy cannot be made at this moment due to high variance and a relatively small number of the available studies. This also implies that the EV-based therapy is not yet ready for clinical application and that further data are needed to design the optimal intervention, create comprehensive experimental protocols, and to incorporate these protocols into clinical setting. Our findings are less clear in several other areas. First, it was not possible to investigate whether a certain EV type or combinations of these are most effective. EVs represent a heterogeneous collection and it remains to be determined which subpopulations confer the protective effects, their target pathways, and the specific molecules they interact with their targets. Also, when designing future studies, several methodological aspects should be taken into consideration: (1) improvement of the study quality (f.ex., by using randomisation into study groups) (2) continuous, growing adherence to the ISEV guidelines, which is needed for standardisation and comparison between studies [66]; (3) use of a gradient of EV doses in animal models; (4) extension of the assessment of kidney function to extrapolate the treatment effect into later CKD stages; and (5) evaluation of EV molecular content at rigorous purity of EV preparations (it was documented that miRNA to EV ratios are low and that in cell culture supernatants or biofluids, a large fraction of miRNAs is present outside EVs [67–70]). Finally, limited data are available on the long-term structural outcomes and safety of EV administration, and about the molecular effects of early versus later dosing of EVs in experimental CKD.

Several limitations of this study should be mentioned. The results of every meta‐analysis are determined by the quality and quantity of the original studies. Our findings are based on a number of heterogeneous studies, interventions, and outcomes, which is inherent in animal research. Most experimental studies in CKD used protocols that differ from model to model and between laboratories, in addition to the heterogeneity inherent in different EV subtypes. Furthermore, a significant proportion of the studies did not report according to ISEV standards. The relatively small number of studies and large heterogeneity also indicates that the conclusions about the effectiveness of EV-therapeutic strategies should be interpreted with caution. Finally, the studies included in this review focused on the protective properties of populations of EVs, that were mainly derived from MSCs and it is not known how these results relate to other EV populations. For example, endothelial cell-EVs were recently shown to attenuate cardiac ischemic injury [71] and they express a protective miRNA signature. All these issues represent areas for future research.

Despite these limitations, our manuscript is the first report that systematically aggregates the data regarding the functional nephroprotective properties of EVs in pre-clinical studies of CKD, connects these data with molecular findings, and summarises the current state of the EV-biomarker field in experimental CKD. Collectively, the included literature demonstrates that the concept of EV-based treatment for CKD, remains a promising one, but that more research is needed regarding standardisation of EV protocols, improving study quality, determining the optimal EV delivery and dosage, and most importantly, understanding the global biological mechanisms of the observed protective effects.

## Supplementary Information

Below is the link to the electronic supplementary material.

Supplementary file1 (PDF 6498 KB)

## Data Availability

The dataset analysed in the manuscript can be obtained from author on reasonable request.
