# Supplementary material for: The Nephroprotective Properties of Extracellular Vesicles in Experimental Models of Chronic Kidney Disease: a Systematic Review
Source: Stem Cell Rev Rep. 2021 Jun 10;18(3):902–32. doi: 10.1007/s12015-021-10189-9 (PMC8942930; doi:10.1007/s12015-021-10189-9)

Supplementary material

The nephroprotective properties of extracellular vesicles in experimental models of chronic kidney disease: a systematic review.

Natalia Nowak[1], Masayuki Yamanouchi[2], Eiichiro Satake [3]

[1]Medical University of Bialystok, Faculty of Medicine, Centre for Bioinformatics and Data Analysis, Bialystok, Poland;

[2]Department of Nephrology and Laboratory Medicine, Faculty of Medicine, Institute of Medical, Pharmaceutical and Health Sciences, Graduate School of Medical Sciences, Kanazawa University, Ishikawa, Japan, Nephrology Center, Toranomon Hospital, Tokyo, Japan

[3]Section on Genetics and Epidemiology, Research Division, Joslin Diabetes Center, Boston, MA, USA, Department of Medicine, Harvard Medical School, Boston, MA, USA

Corresponding author:
Natalia Nowak, Phd
Medical University of Bialystok
Waszyngtona 13 Street
Bialystok, Poland
E-mail: nzuznowak@gmail.com
natalia.nowak@umb.edu.pl

Supplementary Table 1. Appraisal of research methods and risk of bias summary

Study	Concentration	Size	TEM	Protein mrkers	IF	Blinding	Ethics statement	Randomisation	COI
Cambier, 2018 [17]	MF, concentration	Yes (NTA)*	Yes*	Yes*	Yes	-	Yes	-	#
Cantaluppi, 2015 [18]	UC	Yes (NTA)	Yes	Yes	Yes	-	Yes	-	None
Chen, 2019 [11]	UC	Yes (DLS)	Yes	Yes	Yes	-	Yes	Yes	None
Choi, 2015 [19]	UC	-	?	-	Yes	Yes	Yes	Yes	None
Duan, 2020 [20]	UC, MF	Yes (DLS)	Yes	Yes	Yes	-	Yes	-	None
Duan, 2019 [21]	Precipitation	Yes (TEM)	Yes	Yes	Yes	-	Yes	Yes	None
Ebrahim, 2018 [22]	UC	Yes (TEM)	Yes	Yes	Yes	-	Yes	Yes	None
Eirin, 2020 [23]	UC	Yes (NTA)*	Yes*	Yes	Yes	Yes	Yes	-	#
Eirin, 2017 [24]	UC	Yes (NTA)	Yes	Yes	Yes	-	Yes	Yes	None
Grange, 2019 [25]	UC, MF	Yes (NTA)	-	MSC-EV markers only	Yes	-	Yes	Yes	#
He, 2012 [26]	UC	Yes (TEM)	Yes	-	Yes	Yes	Yes	Yes	-
He, 2015 [27]	UC	Yes (TEM)	Yes*	-	Yes	Yes	Yes	Yes	None
Ji, 2020 [28]	UC, MF	Yes (NTA)	Yes	Yes	Yes	-	Yes	-	None
Jiang, 2016 [29]	UC, MF, DG	Yes (TRPS)	Yes	Yes	Yes	Yes	Yes	Yes	None
Jin, 2019 [30]	Immun-P, UF	Yes (NTA)	Yes	Yes	Yes	-	Yes	-	None
Jin, 2020 [31]	Immun-P	Yes (TEM)	Yes	Yes	Yes	-	n.a.	-	None
Kholia, 2018 [32]	DG-UC	Yes (NTA)	Yes	Yes	Yes	Yes	Yes	-	-
Kholia, 2020 [33]	UC, MF	Yes (NTA)	Yes	Yes	Yes	-	Yes	-	None
Lindoso, 2020 [34]	UC	Yes (NTA)	Yes	Yes	Yes	Yes	Yes	Yes	None
Nagaishi, 2016 [35]	UC	Yes (TEM)	Yes	Yes	Yes	-	Yes	-	None
Ramirez-Bajo, 2020 [13]	UC, MF	Yes (NTA)	Yes	Yes	Yes	-	Yes	Yes	#
Sedryakyan, 2017 [36]	UC	Yes (NTA)	-	Yes	Yes	Yes	Yes	-	None
Shi, 2020 [37]	UC	Yes (NTA)	Yes	Yes	Yes	-	Yes	Yes	None
Song, 2020 [15]	UC	Yes (NTA)*	Yes*	Yes	Yes	-	Yes	-	#
Van Koppen, 2012 [12]	DG -UC	-	-	-	Yes	-	Yes	-	#
Wang, 2020 [38]	Column method	-	-	MSC-EV markers only	Yes	-	Yes	Yes	None
Wang, 2015 [39]	UC	-	-	MSC-EV markers only	Yes	Yes	Yes	Yes	None
Wang, 2016 [40]	Precipitation	?	?	?	Yes	-	Yes	-	None
Wang, 2019 [41]	UC, MF	Yes (NTA)	-	Yes	Yes	Yes	Yes	-	None
Yang, 2019 [42]	UC	Yes (TEM)	Yes	-	Yes	Yes	Yes	-	None
Zhang, 2019 [43]	Serial centrifugation	Yes (TEM)	-	Yes	Yes	-	Yes*	-	None
Zhang, 2019 [44]	UC	Yes (TEM)	-	MSC-EV markers only	Yes	-	Yes	Yes	None
Zhao, 2020 [45]	UC	Yes (NTA)	Yes	Yes	Yes	Yes	Yes	Yes	#
Zhong, 2019 [46]	UC	Yes (NTA)	Yes	MSC-EV markers only	Yes	-	Yes	Yes	None
Zou, 2018 [47]	UC	Yes (NTA)	Yes	Yes	Yes	-	Yes	Yes	None

Abbreviations: NTA – Nanoparticle tracking analysis, UC – Ultracentrifugation, TEM – Transmission Electron Microscopy, TRPS- Tunable Resistive Pulse Sensing, MF – Microfiltration, asterisk indicates that the data had been reported in the previous references by the same authors, minus indicates that information not available, n.a. indicates ethic statement was likely not required (in vitro study), # indicates that conflict of interest (COI) is reported but does not affect, in our interpretation, the study results.

References:

1) de Couto, G., Gallet, R., Cambier, L., Jaghatspanyan, E., Makkar, N., Dawkins, J. F., Berman, B. P., & Marbán, E. (2017). Exosomal MicroRNA Transfer Into Macrophages Mediates Cellular Postconditioning. Circulation, 136(2), 200–214.

2) Ibrahim, A. G., Cheng, K., & Marbán, E. (2014). Exosomes as critical agents of cardiac regeneration triggered by cell therapy. Stem cell reports, 2(5), 606–619.

3) Collino, F., Bruno, S., Incarnato, D., Dettori, D., Neri, F., Provero, P., Pomatto, M., Oliviero, S., Tetta, C., Quesenberry, P. J., & Camussi, G. (2015). AKI Recovery Induced by Mesenchymal Stromal Cell-Derived Extracellular Vesicles Carrying MicroRNAs. Journal of the American Society of Nephrology : JASN, 26(10), 2349–2360.Table 2.

4) Eirin, A., Zhu, X. Y., Puranik, A. S., Tang, H., McGurren, K. A., van Wijnen, A. J., et al. (2017). Mesenchymal stem cell-derived extracellular vesicles attenuate kidney inflammation. Kidney international, 92(1), 114–124.

Supplementary table 2. Details of the design of the studies that focused on genetically modified EVs/miRNA effector molecules. Characteristics of the EV intervention, disease/animal model, time-points evaluated, and main study findings are outlined.

CKD model	Study	EV/ miRNA source	Dose	Adm-route	Dose	EV size	EV markers	Species/Model	Sex	EA	End	Main findings
UUO	Wang, 2019	miR-29-Satellite cells (m)	M	IM	n.a.	87-93 nm (NTA)	TSG101	C57BL/6J mouse (UUO)	M	1w	2w	miR-29-EVs attenuated renal fibrosis and improve kidney function in UUO mice.
UUO	Zhang, 2019	EKC (h) miR-26a-EVs	S	IM	n.a.	30-500 nm (NTA)	TSG101	C57BL/6J mouse (UUO)	M	1d	2w	miR-26a-EVs improved kidney fibrosis in UUO mice.
UUO	Wang, 2016	let7c-MSCs (h)	n.a.	n.a.	-	n.a.	?	In vitro (mouse TECs, TGF-β)	n.a.	-	-	let-7c-EVs inhibited fibrosis gene expression.
DM	Jin, 2020	MSC(m)-EVs-miR-215	n.a.	n.a.	-	87-93 nm (NTA)	CD9, CD63, CD81	In vitro (mouse podocyte, glucose)	n.a.	-	-	EVs and EV-miR-215 inhibited fibrosis gene expression.

Abbreviations: Cell of EV origin: EKC; embryonic kidney cell, MSC; mesenchymal stem cell. NTA; nanoparticle tracking analysis, TSG101; Tumor Susceptibility 101. CKD model: DM; diabetes, UUO; unilateral ureteral obstruction. TEC; tubular epithelial cell, TGF-β1; transforming growth factor β.

Supplementary Table 3. Summary of the studies which evaluated the protective effect of extracellular vesicles (EVs): study reference, cell of origin of EVs, injury model, effector molecules mediating protection, and the involvement in CKD mechanism are specified. The effect of EVs is represented: red indicates a reduction, blue indicates an elevation.

				Fibrosis			Cell damage				Inflammation				Stress
Study	CKD	Cell	Mediator	GS	IF	Fibrosis Markers	Cell Death	Caspase	ACR	KIM-1 NGAL	WBC	M1 to M2	TNF, IL-6	IL-10	Oxidative stress
Chen, 2019	**UUO**	MSC	GDNF												
Choi, 2015	**UUO**	MSC													
He, 2015	**UUO**	MSC	miRNA array												
Ji, 2020	**UUO**	MSC	CK1δ, β-TRCP												
Shi, 2020	**UUO**	MSC	MFG-E8												
Wang, 2020	**UUO**	MSC	miR-294, miR-133												
Wang, 2015	**UUO**	MSC	miRNA array												
Wang, 2016	**UUO**	MSC	let-7c												
Wang, 2019	**UUO**	SC	miR-29												
Yang, 2019	**UUO**	EPC													
Zhang, 2019	**UUO**	EKC	miR-26a												
Cantaluppi, 2015	**Toxic**	EPC	CD55,CD59, CFH miR-126, miR-296												
Kholia, 2018	**Toxic**	HLSC													
Matsakura, 2019	**Toxic**	MSC													
Ramirez, 2020	**Toxic**	MSC													
Zhang, 2019	**Toxic**	MSC													
He, 2012	**Nx**	MSC													
Koppen, 2012	**Nx**	MSC													
Cambier, 2018	**HT**	CPC	Y4 RNA												
Eirin, 2017	**HT**	MSC	IL10 mRNA												
Eirin, 2020	**HT**	MSC	RNA-seq												
Lindoso, 2020	**HT**	MSC													
Song, 2020	**HT**	MSC	RNA-seq												
Zhao, 2000	**HT**	MSC	miR-532-5p												
Zou, 2018	**HT**	STC	mito-DNAs												
Sedryakyan, 2017	**Alport**	AFSC	VEGFR1, miRNAs												
Duan, 2000	**DM**	MSC	miR-26a-5p												
Duan, 2019	**DM**	uSC	miR-16-5p												
Ebrahim, 2018	**DM**	MSC													
Grange, 2019	**DM**	MSC, HLSC	miRNA array												
Jiang, 2016	**DM**	uSC													
Jin, 2019	**DM**	MSC	miR-486												
Jin, 2020	**DM**	MSC	miR-215-5p												
Nagaishi,2016	**DM**	MSC													
Zhong, 2019	**DM**	MSC	miR-451a												

Abbreviations: GS; percent glomerular sclerosis, IF; percent tubulointerstitial fibrosis ACR albumin to creatinine ratio; M1 to M2 macrophage ratio, IL-10; interleukin-10, tubular markers: KIM-1; kidney injury molecule-1, NGAL; neutrophil gelatinase-associated lipocalin, WBC; White blood cell infiltrates, NGAL; Neutrophil gelatinase-associated lipocalin,WBC; White blood cell infiltrates, Cell of EV origin: AFSC; amniotic fluid stem cell, CPC; cardiac progenitor cell, EKC; embryonic kidney cell, EPC; endothelial progenitor cell, HLSC; liver stem cell, MSC; mesenchymal stem cell, SC; satellite cell, STC; STC-like cell, uSC; urine stem cell.

Supplementary figure 1; Standardized mean difference (SMD) in glomerular filtration rate (GFR). Data represent SMD calculated for treaded versus non-treated comparisons of all records, with the 95% confidence interval (95% CI). RE, random effect

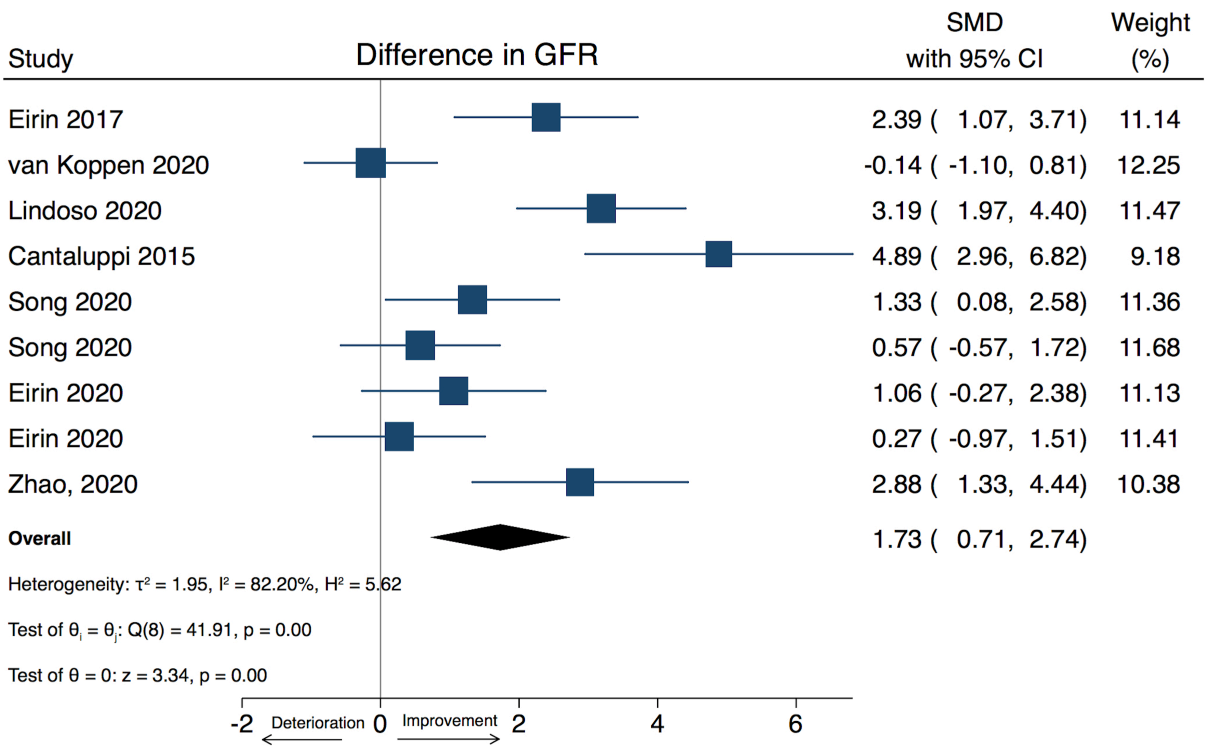

Supplementary figure 2; Subgroup analyses: standardized mean difference (SMD) in (A) serum creatinine and (B) urea. Data represent SMD based on EV intervention-related and model related factors with the 95% confidence interval (95% CI). RE, random effect.

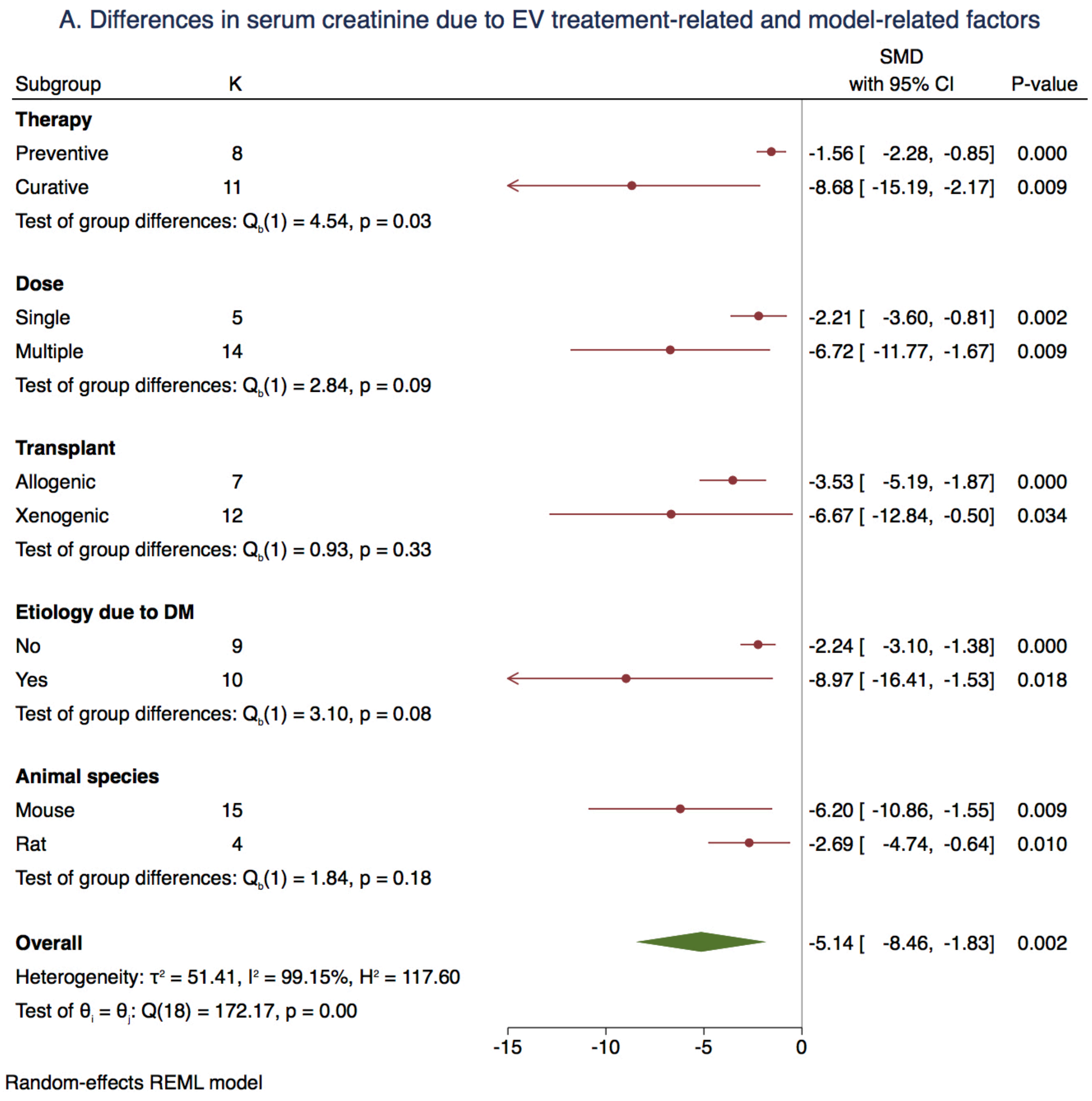

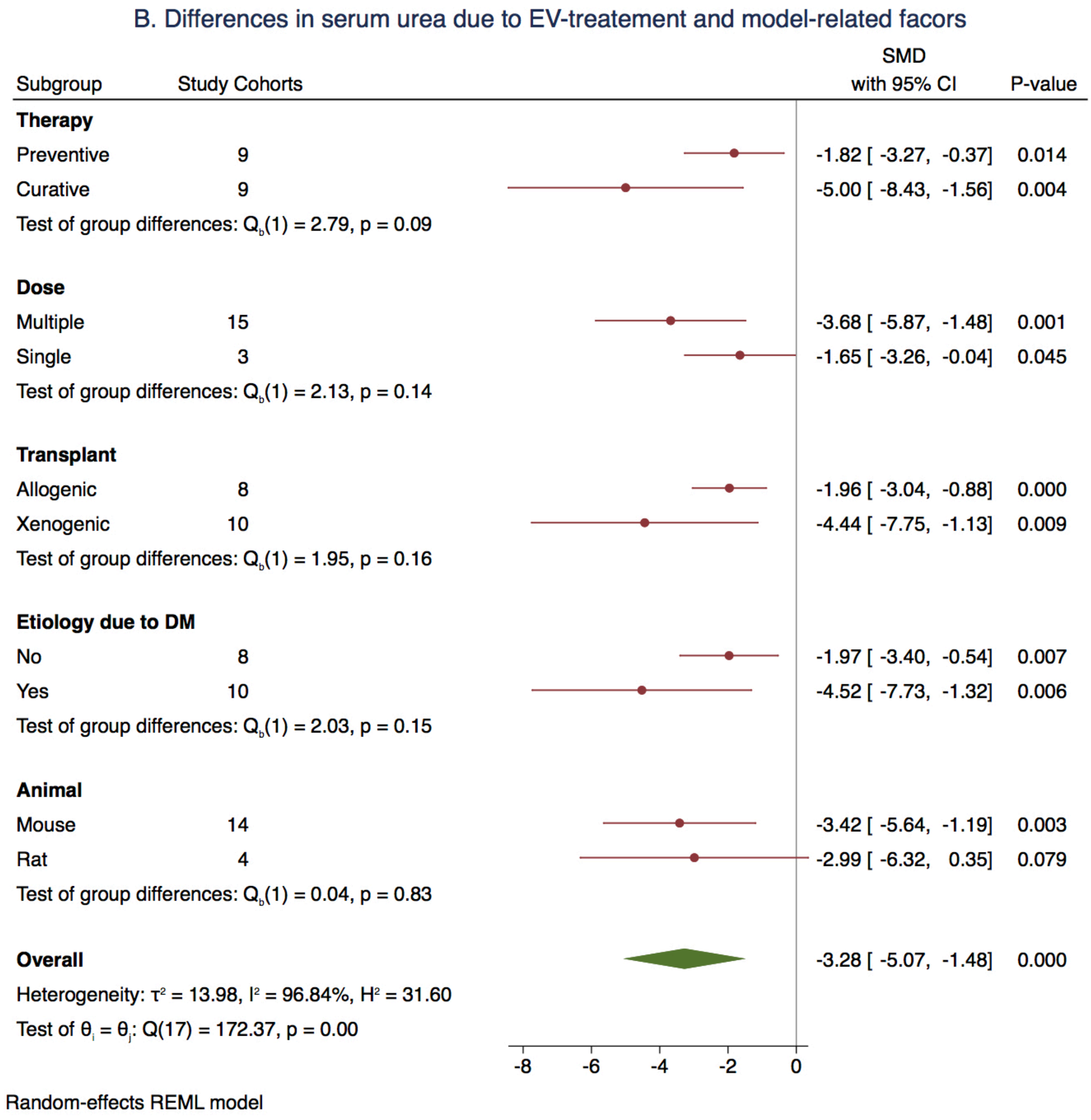

B. Differences in serum urea due to EV-treatement and model-related facors
Subgroup
Study Cohorts
SMD with 95% CI
P-value
Therapy
Preventive 9 -1.82 [-3.27, -0.37] 0.014
Curative 9 -5.00 [-8.43, -1.56] 0.004
Test of group differences: $Q_b(1)$ = 2.79, p = 0.09
Dose
Multiple 15 -3.68 [-5.87, -1.48] 0.001
Single 3 -1.65 [-3.26, -0.04] 0.045
Test of group differences: $Q_b(1)$ = 2.13, p = 0.14
Transplant
Allogenic 8 -1.96 [-3.04, -0.88] 0.000
Xenogenic 10 -4.44 [-7.75, -1.13] 0.009
Test of group differences: $Q_b(1)$ = 1.95, p = 0.16
Etiology due to DM
No 8 -1.97 [-3.40, -0.54] 0.007
Yes 10 -4.52 [-7.73, -1.32] 0.006
Test of group differences: $Q_b(1)$ = 2.03, p = 0.15
Animal
Mouse 14 -3.42 [-5.64, -1.19] 0.003
Rat 4 -2.99 [-6.32, 0.35] 0.079
Test of group differences: $Q_b(1)$ = 0.04, p = 0.83
Overall -3.28 [-5.07, -1.48] 0.000
Heterogeneity: τ^2 = 13.98, I^2 = 96.84%, H^2 = 31.60
Test of $\theta_i = \theta_j$: Q(17) = 172.37, p = 0.00
-8 -6 -4 -2 0
Random-effects REML model

Supplementary figure 3; Sensitivity analysis: **s**tandardized mean difference (SMD) in (A) serum creatinine and (B) urea for all studies in animal CKD. Data represent SMD calculated for treaded versus non-treated comparisons of all records, with the 95% confidence interval (95% CI). RE, random effect.

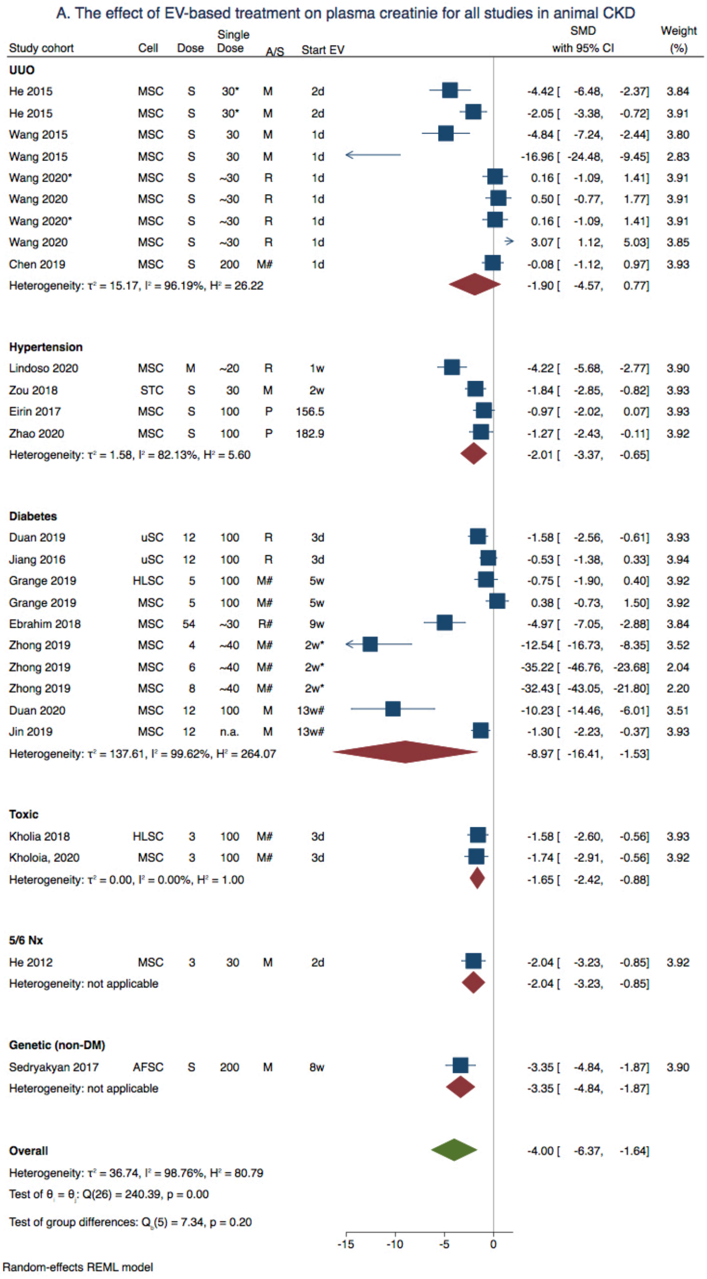

B. The effect of EV-based treatement on plasma urea for all studies in CKD

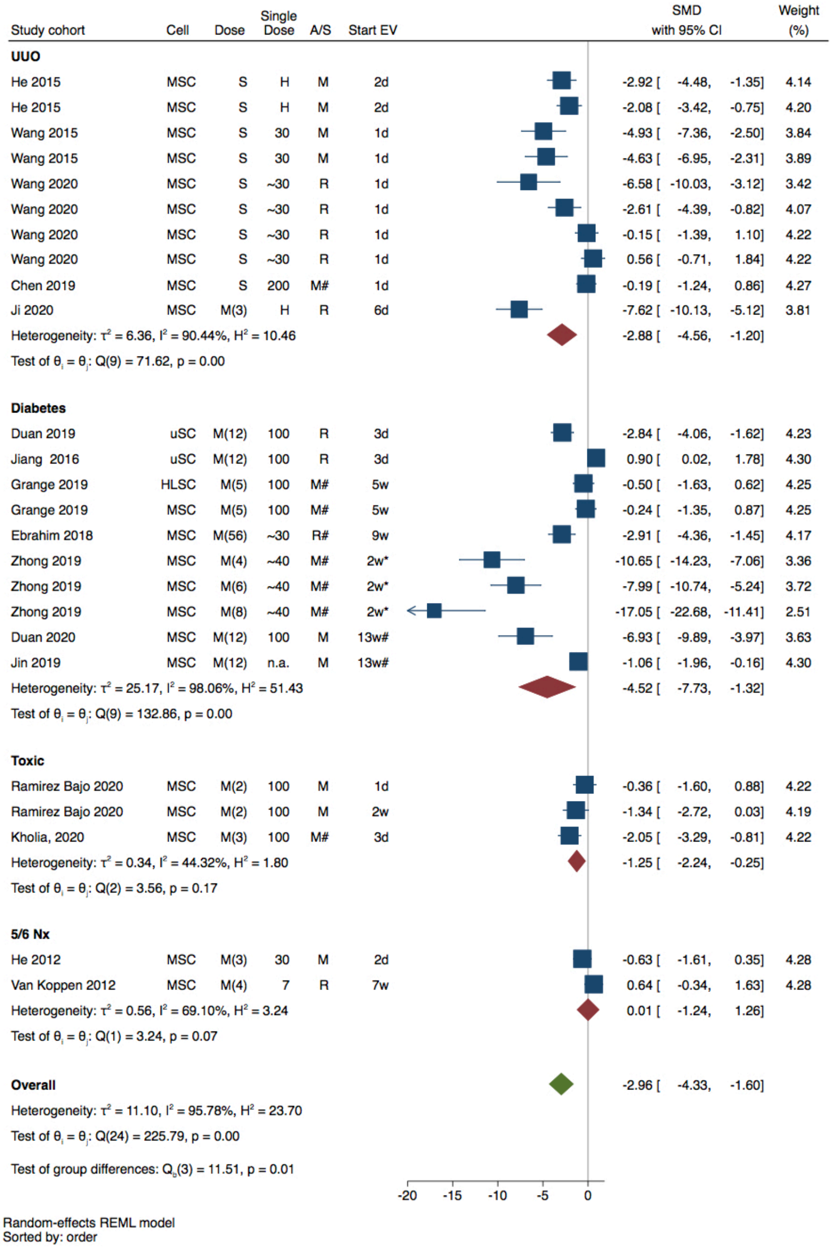

Supplementary figure 4; Subgroup analyses conducted for all study cohorts: standardized mean difference (SMD) in (A) serum creatinine and (B) urea. Data represent SMD calculated based on different factors, with the 95% confidence interval (95% CI). RE, random effect.

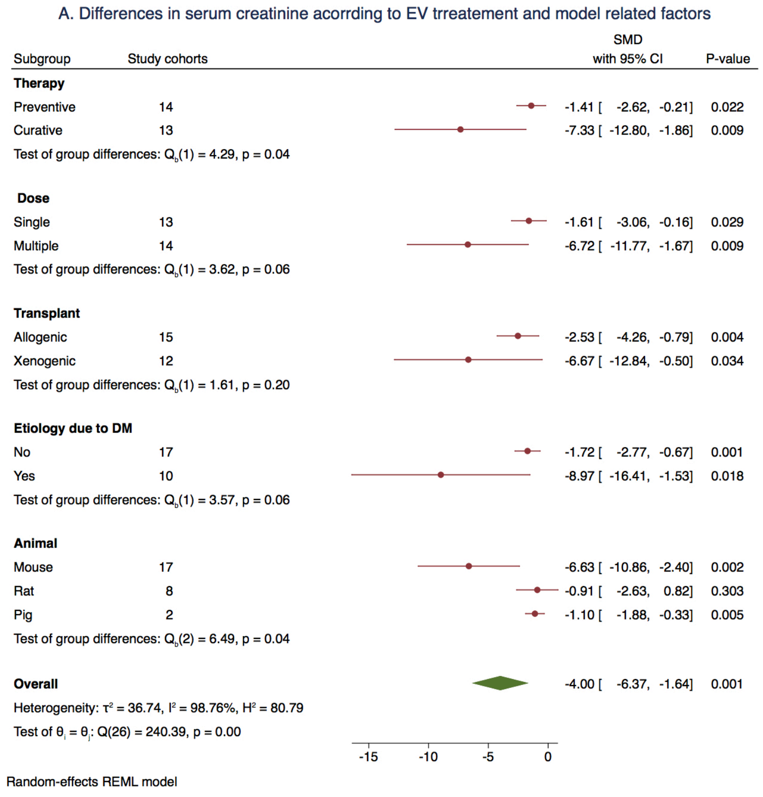

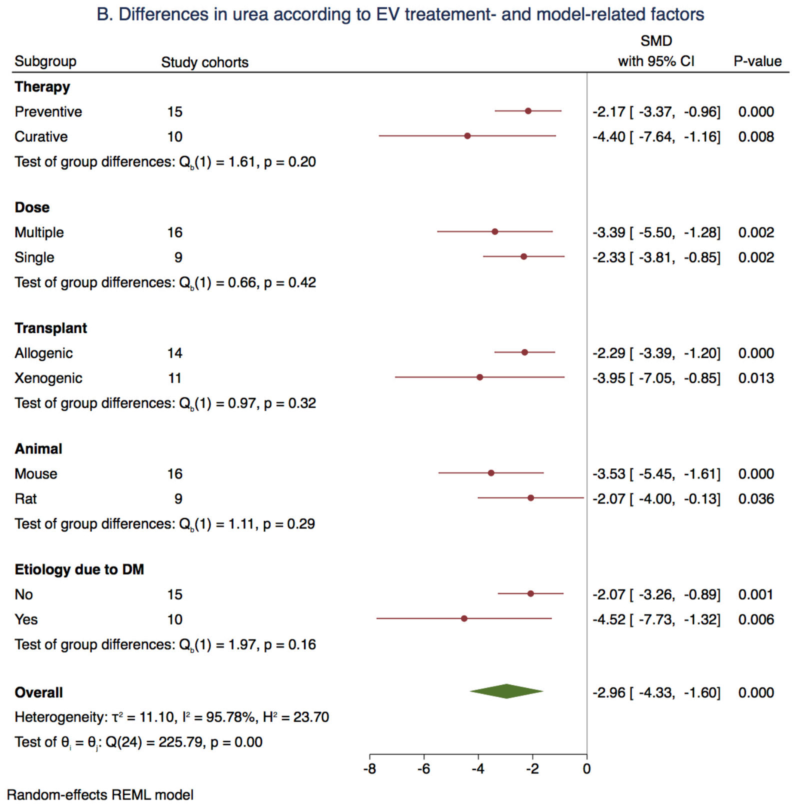

Supplement: Supplementary file 1 — Supplementary file1 (PDF 6498 KB) [file 12015_2021_10189_MOESM1_ESM.pdf]
